# Satellite Observations Reveal a Positive Relationship Between Trait‐Based Diversity and Drought Response in Temperate Forests

**DOI:** 10.1111/gcb.70059

**Published:** 2025-02-03

**Authors:** Isabelle S. Helfenstein, Joan T. Sturm, Bernhard Schmid, Alexander Damm, Meredith C. Schuman, Felix Morsdorf

**Affiliations:** ^1^ Remote Sensing Laboratories, Department of Geography University of Zurich Zurich Switzerland; ^2^ Eawag, Swiss Federal Institute of Aquatic Science and Technology, Surface Waters – Research and Management Duebendorf Switzerland; ^3^ Department of Chemistry University of Zurich Zurich Switzerland

**Keywords:** biodiversity–ecosystem functioning (BEF), ecological monitoring, functional diversity, plant traits, remote sensing, Sentinel‐2

## Abstract

Climate extremes such as droughts are expected to increase in frequency and intensity with global change. Therefore, it is important to map and predict ecosystem responses to such extreme events to maintain ecosystem functions and services. Alongside abiotic factors, biotic factors such as the proportion of needle and broadleaf trees were found to affect forest drought responses, corroborating results from biodiversity–ecosystem functioning (BEF) experiments. Yet it remains unclear to what extent the behavior of non‐experimental systems at large scales corresponds to the relationships discovered in BEF experiments. Using remote sensing, the trait‐based functional diversity of forest ecosystems can be directly quantified. We investigated the relationship between remotely sensed functional richness and evenness and forest drought responses using data from temperate mixed forests in Switzerland, which experienced an extremely hot and dry summer in 2018. We used Sentinel‐2 satellite data to assess aspects of functional diversity and quantified drought response in terms of resistance, recovery, and resilience from 2017 to 2020 in a scalable approach. We then analyzed the BEF relationship between functional diversity measures and drought response for different aggregation levels of richness and evenness of three physiological canopy traits (chlorophyll, carotenoid/chlorophyll ratio, and equivalent water thickness). Forest stands with greater trait richness were more resistant and resilient to the drought event, and the relationship of trait evenness with resistance or resilience was hump‐shaped or negative, respectively. These results suggest forest functional diversity can support forests in such drought responses via a mixture of complementarity and dominance effects, the first indicated by positive richness effects and the second by negative evenness effects. Our results link ecosystem functioning and biodiversity at large scales and provide new insights into the BEF relationships in non‐experimental forest ecosystems.

## Graphical abstract

The relationship between functional diversity (richness, evenness) and drought response (resistance, recovery, resilience) during the severe 2018 drought in Switzerland was analyzed using satellite‐derived data. The Sentinel‐2 data revealed that forests with greater canopy trait diversity showed higher resistance and resilience to drought. These results align with experimental findings that biodiversity can strengthen forests' drought response and demonstrate the link between functional diversity and forest resilience under drought stress in non‐experimental forest ecosystems at landscape scales.

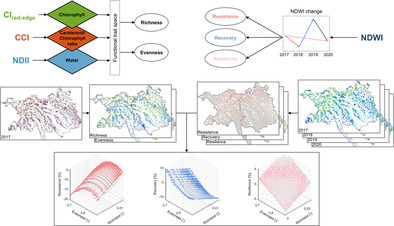

## 1 Introduction

Global climate change is expected to increase both the frequency and intensity of climate extremes (Smith 2011), and so it is of growing importance to study ecosystem responses to these extremes. Rising temperatures due to global change and related evapotranspiration dynamics are predicted to amplify drought stress in Europe (Jacob et al. 2014) and increasingly challenge the capacity of ecosystems to maintain high levels of ecosystem functioning (EF). Understanding how changing environmental conditions influence processes across levels of ecological organization is critical for predicting EF and impacts on ecosystem service provisioning (Suding et al. 2008). For example, the extreme 2018 summer drought in central Europe caused unprecedented forest mortality, highlighting the need for a monitoring network to track climate change impacts (Schuldt et al. 2020).

Drought occurs through a deficit in ecosystem water availability below a vulnerability threshold that affects ecosystem services (Crausbay et al. 2017). Drought responses can be divided into resistance—performance during drought, recovery—performance after drought, and resilience—the similarity of the performance before and after the event (Lloret, Keeling, and Sala 2011). Multiple abiotic factors may influence ecosystem responses to drought, such as topography, soil, and weather conditions (Rita et al. 2020). Recent studies suggest that alongside multiple abiotic factors such as topography, biotic factors such as the proportion of needle and broadleaf trees are explanatory variables for drought responses (Sturm et al. 2022).

Evidence from experiments shows that biodiversity enhances stability, the ability of ecosystems to maintain functioning under stressful environmental conditions (Isbell et al. 2015). Studies focusing on resistance and resilience found that forest stands containing multiple species were less affected by drought than mono‐specific stands (Lebourgeois et al. 2013), whereas others found no differences in drought responses of trees with different neighboring species (Forrester et al. 2016). There is growing recognition of the importance of trait‐based diversity to understand the influence of biodiversity on forest functioning, and trait diversity is expected to promote EF (Ruiz‐Benito et al. 2014). Rather than the number of species alone, the dissimilarity of functions can positively impact forest drought responses (Cadotte, Carscadden, and Mirotchnick 2011). This dissimilarity of functions can be represented by, for example, leaf ecophysiological traits representing the leaf economics spectrum (Díaz et al. 2016) or morphological traits, such as tree height or wood density (Gazol and Camarero 2016). It is conceivable that a particular combination of traits causes biodiversity effects such as resistance and resilience to stress; it is, however, not clear which trait combination might link to biodiversity effects and whether they are consistent across different environmental and community contexts, including multiple species mixtures (Huang et al. 2018; Luo et al. 2023). In one study, functional diversity in tree height, wood density, seed mass, and seed dispersal did not relate to drought responses (Espelta et al. 2020). Recent evidence suggests that biodiversity–ecosystem functioning (BEF) relationships in forest ecosystems are modulated by differences in leaf traits (Feng et al. 2022). In their analysis of forest drought responses across Switzerland, Sturm and colleagues (Sturm et al. 2022) found that mixed stands of broadleaf and needle trees could cope better with drought than pure broadleaf or needle stands, but they could not measure functional or taxonomic diversity at a finer scale than the difference between angiosperms and gymnosperms.

Trait‐based diversity is a widely used approach for quantifying the functional contributions of individuals or species to ecosystem properties (Cadotte, Carscadden, and Mirotchnick 2011). Thus, sampled objects (pixels, individuals, and species) can be classified using traits, defining these objects' functional roles within communities and responses to environmental variables (Petchey and Gaston 2006). With increasing functional diversity, a greater range of functional trait values is present, providing opportunities for efficient resource use (Díaz and Cabido 2001). Trait‐based diversity can be quantified with diversity metrics describing the multidimensional trait space.

Predicting how ecosystems and the services they provide will respond to accelerating environmental change will benefit from comprehensive, globally consistent, and repeated data on the patterns and dynamics of functional diversity (Jetz et al. 2016). Remote sensing (RS) allows the diversity of temperate forest ecosystems to be quantified directly at landscape scales through physiological canopy traits (Schneider et al. 2017; Helfenstein et al. 2022), which is particularly relevant as resource management decisions are generally made at these scales (Nelson et al. 2009). While the spatial resolution of satellite sensors may limit the detection of subtle trait variations across species and individuals (Petibon et al. 2021; Schneider et al. 2017), RS complements detailed but spatially limited field measurements by providing spatially contiguous and multi‐scale information on certain traits (e.g., pigments and water content) and their dynamics throughout the phenological cycle, provided sufficient spectral and temporal resolution (Homolová et al. 2013). However, satellite‐based observations primarily capture the uppermost canopy layers, while lower forest strata and soil background are not consistently represented (Damm et al. 2020), likely reducing information on 3D canopy variation and below‐ground variables. Despite these limitations, canopy trait‐based diversity is considered an effective measure for mapping biodiversity and detecting its effects on EF using RS data (Jetz et al. 2016; Zheng et al. 2023), without requiring additional information on tree species composition.

Beyond initial studies such as the ones mentioned above (Sturm et al. 2022; Schneider et al. 2017; Zheng et al. 2023), the sensitivity of satellite‐derived measures of trait‐based functional diversity and the linkage between these and EF in general or ecosystem drought responses, in particular, have not been rigorously assessed (Jetz et al. 2016). Filling this gap could advance our understanding of climate change impacts on forest ecosystems and pave the way toward large‐scale assessment and long‐term forest diversity and resilience monitoring. Here, we used Sentinel‐2‐derived trait‐based functional diversity measured at landscape scales in 2017, and Sentinel‐2‐derived drought response assessments using changes in the normalized difference water index [NDWI, a measure of forest canopy water content (Lloret, Keeling, and Sala 2011; Sturm et al. 2022)] from 2017 to 2020, to study the link between trait‐based functional diversity as a biodiversity measure and drought response as an EF measure for the cantons of Aargau and Zurich on the Swiss Plateau. We chose this area because abiotic factors (e.g., topography‐related air temperature and illumination, precipitation) are far less variable across the Swiss plateau than throughout the entire country (Sturm et al. 2022), allowing us to focus on relationships between variation in tree diversity and variation in forest drought response. To account for the remaining abiotic variability in the study region, we divided the region into 21 geographic subregions.

The summer weather in 2018 in central Europe was dominated by large precipitation deficits, high temperatures, and sunny conditions over large areas (MeteoSchweiz 2018). In Switzerland, the mean precipitation between April and September was just above 500 mm (the lowest since 1962) and the mean temperature was the highest since measurements started in 1864 (MeteoSchweiz 2018). In Swiss temperate forests, the drought resulted in early wilting, decreased forest health, and widespread tree mortality (Sturm et al. 2022). Secondary drought effects followed, for example, in 2019, the amount of wood infested by bark beetles (*Ips typographus*) in Switzerland reached over one million m^3^ for the first time since 2005 (Stroheker, Forster, and Queloz 2020).

We compared the changes in NDWI between pre‐drought conditions in 2017, drought conditions in 2018, and post‐drought conditions in 2019 and 2020. All four years had dry and warm summer conditions compared to the long‐term reference period (Rakovec et al. 2022), but the severity of the drought in 2018 contrasts with the other years (Sturm et al. 2022). 2018 was marked by the most severe drought of the analyzed years, with the highest summer temperature maxima and prolonged periods of below‐average precipitation. These conditions were described in detail by Sturm et al. (2022) and are further illustrated in Figure S1, using precipitation and temperature data from MeteoSwiss (Scherrer et al. 2022, Figure S1). Although warm, 2019 and 2020 experienced more precipitation, resulting in relatively favorable growing conditions (MeteoSchweiz 2020, 2021). In particular, 2020 was characterized by a prolonged dry spell in spring and moderate heatwaves later in the season. Precipitation increased toward the end of the summer, alleviating drought impacts (MeteoSchweiz 2021).

The aim of this study was to investigate the relationships between trait‐based functional diversity metrics and forest drought responses using Sentinel‐2‐derived canopy traits. We aim to provide insights into how satellite‐based functional diversity influences forest stability under drought conditions, advancing our understanding of BEF relationships at landscape scales. We focused on how forest drought responses (resistance, recovery, and resilience) across the years of 2017–2020 were related to trait‐based functional diversity metrics (richness and evenness).

We used three leaf traits that can be assessed at the canopy level using spectral indices: chlorophyll content (CHL), carotenoid/chlorophyll ratio (CCR), and equivalent water thickness (EWT) (Helfenstein et al. 2022; Schneider et al. 2017). The two diversity metrics we used, richness and evenness, are commonly used in BEF research (Mammola et al. 2021). We calculated two complementary metrics to gain distinct insights into the separate aspects of diversity and their relationships with drought responses. Richness relates to the hypervolume of the trait space occupied by a community of a certain unit area at a certain time. The larger the richness, the greater the extent of the hypervolume, measured, for example, using convex hulls (Villéger, Mason, and Mouillot 2008). Functional richness is different from other functional diversity measures, like Rao's *Q*, that use mean differences between species and which are therefore independent of species richness (Huang et al. 2018). Here we prefer functional richness as a measure because it relates to species richness, whose effects are commonly studied in field‐based BEF research (Liu et al. 2024). Evenness measures the regularity of the observations' distribution within the hypervolume (Mammola et al. 2021). If used with species diversity metrics, evenness refers to the similarity of species abundance values independent of species number. Conceptually, evenness reflects how equally different functional trait values are distributed in a community (Villéger, Mason, and Mouillot 2008). When the occupation of the hypervolume is skewed toward some specific trait values, then those traits are dominant within the community and evenness is low (Mammola et al. 2021). Conversely, high evenness (i.e., more uniform occupation of the hypervolume) implies weak or no dominance of specific trait values and thus species carrying those trait values.

Relating functional richness and evenness to species richness and evenness suggests that with high richness, it is possible to have complementarity and selection (i.e., dominance) effects as defined by the additive partitioning method of biodiversity net effects (Loreau et al. 2001). In a forest with high realized evenness, complementarity effects strongly contribute to biodiversity net effects, while dominance effects necessarily reduce realized evenness. At intermediate levels of realized evenness (and high richness), both effects can contribute positively to net biodiversity effects. Therefore, we hypothesized a positive relationship between functional richness and drought response and a hump‐backed relationship between evenness and drought response. Furthermore, whereas richness is related to the size of the hypervolume, evenness can be high even within a small hypervolume in trait space, that is, low richness. Thus, we expected the relationship between functional richness and drought response to be stronger than the relationship between functional evenness and drought response.

## 2 Materials and Methods

### 2.1 Study Area

**FIGURE 1:**
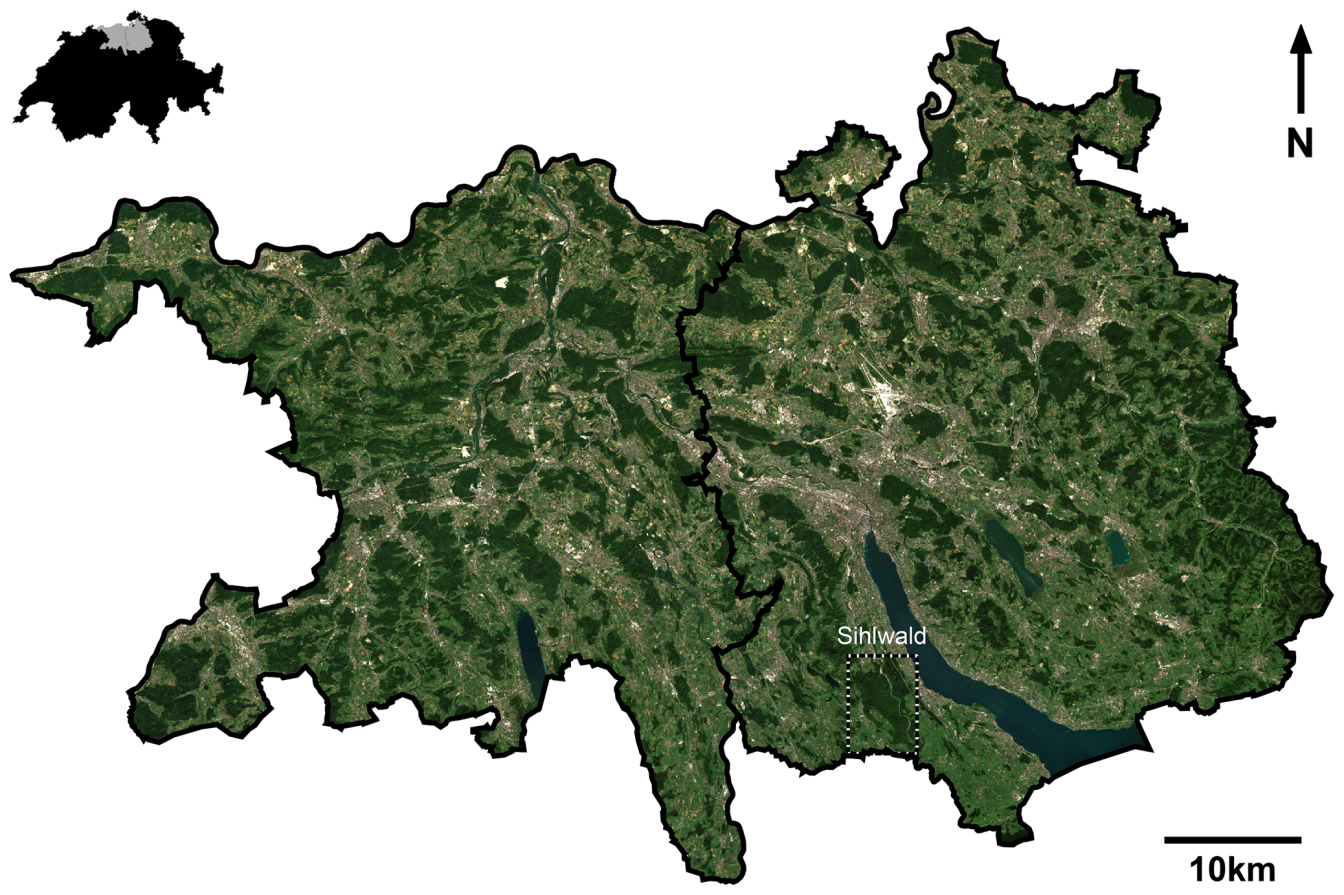
Study area of canton Aargau (west) and canton Zurich (east) and location in Switzerland (top left). Highlighted on the map is the Sihlwald site, where we validated the drought response results. The true color composite shows the study area in summer 2017, based on June/July Sentinel‐2 data. Map lines delineate study areas and subregions and do not necessarily depict accepted national boundaries.

The study area comprises the cantons Aargau and Zurich in Switzerland (Figure 1). The cantonal borders in Figure 1 are based on swissBOUNDARIES3D by swisstopo (2021). Both cantons are located on the northern central plateau, subject to different forest management practices, containing different forest communities. The canton Aargau has a total area of 1403.80 km^2^, of which 35% or 490.70 km^2^ is forested. The main tree species in canton Aargau are European beech ( *Fagus sylvatica* ) with 32% of the cantonal stocks, followed by Norway spruce ( *Picea abies* ) with 26%, silver fir ( *Abies alba* ) with 14%, and sycamore maple ( *Acer pseudoplatanus* ) with 5% (Departement Bau, Verkehr und and Umwelt 2018). The canton Zurich covers an area of 1728.87 km^2^, of which forests cover 29.1% or 503.73 km^2^. The main tree species in canton Zurich are *P. abies* , with 35% of the cantonal stocks, *F. sylvatica* with 24%, *A. alba* , with 12%, and ash ( *Fraxinus excelsior* ) with 8% (Baudirektion Kanton Zürich 2020).

We grouped the forests in the study area according to the intersection of cantonal forest districts and geographical regions into 21 subregions. The subdivision of Switzerland into geographical regions was based on similar ecological characteristics (BAFU 2004, 2022). These geographical regions were the eastern and western Swiss plateau, pre‐Alps, Rhine plains, and Jura mountains. The territorial authority of the cantonal forest service regulates forest districts (AGIS 2023). The forest district data were provided by the cantons (AGIS 2023; GIS‐ZH 2019a). Aargau is divided into four and Zurich into seven forestry districts. Management can be assumed to be similar in one district but might differ between districts. The intersection of geographical regions and forestry districts resulted in 21 subregions with forested areas between 3.5 and 100 km^2^.

### 2.2 Satellite Data

We used a composite of Sentinel‐2 data from three dates in June/July 2017 to generate the diversity maps, that is, Sentinel‐2A images from June 19th and 26th and Sentinel‐2B data from July 4th. Monthly composites from August in the Years 2017–2020 were used to assess the drought response (see Table 1). In August, the drought impacts should be at their full strength, whereas the senescence due to the natural phenological cycle is still absent (MeteoSchweiz 2018). We ensured that the assessments of diversity and drought response were based on independent observations from the independent times of acquisition.

**TABLE 1:** Acquisition dates (left) and sensor type (Sentinel‐2A/B, right) of the satellite data used for the composites to calculate the diversity data (June/July 2017) and the drought response data (August 2017–2020) to create the drought response maps.

Diversity data		Drought response composite data							
June–July 2017		August 2017		August 2018		August 2019		August 2020	
Date	Sensor	Date	Sensor	Date	Sensor	Date	Sensor	Date	Sensor
June 19	A	August 15	A	August 03	A	August 08	A	July 30	A
June 26	A	August 18	A	August 05	B	August 18	A	August 07	B
July 04	B	August 23	B	August 20	A	August 25	A	August 09	A
		August 25	A	August 23	A	August 28	A	August 12	A
		August 30	B	August 28	B	August 30	B	September 03	B

### 2.3 Satellite Data Pre‐Processing

All data were collected using ESA's Scihub and atmospherically corrected using Sen2Cor v.2.9.0 in the ESA Sentinel Application Platform SNAP v9.0. We derived all Sentinel‐2 bands available in 10‐ or 20‐m native spatial resolution. The 10‐m bands were resampled to 20 m using mean resampling.

In all images, we flagged all pixels with < 5% reflectance in band B2 (blue) and > 15% in band B8A (NIR) as cloud‐ and cloud‐shadow‐free, following the approach of Sturm et al. (2022). Additionally, we applied the cantonal polygon forest masks available in LV95 reference system and warped them using gdal to match the projection of the Sentinel‐2 data in WGS 84/UTM 32N (AGIS 2019; GIS‐ZH 2019b). To calculate forest traits in June/July 2017, we excluded pixels covering forest gaps, dead canopies, and shadows to tailor the assessment of canopy traits on living forest canopies only. We therefore derived a forest mask for the scene in June/July 2017, which was then applied to all composites. We set a threshold for the normalized difference vegetation index (NDVI) (bands B4 and B8A) within the forest area. We calculated a median outlier for the forested area, resulting in NDVI thresholds of 0.795 for June 19, 0.8003 for June 26, and 0.81 for July 4, 2017. Lastly, we applied shadow masks based on the bands B6 and B12, excluding the darkest pixels in these bands, defined as median outliers from the overall distribution (Rüfenacht, Fredembach, and Süsstrunk 2013). We calculated three forest maps based on the three acquisitions in June/July 2017. Pixels needed to be valid in two out of three images to be included in the final forest mask using a mean calculation. The resulting forest mask contained 2,293,752 valid pixels and covered a total forest area of 917.5 km^2^.

### 2.4 Leaf Ecophysiological Traits at Canopy Level

Trait‐based functional diversity from RS can be derived for ecophysiological, morphological, or phenological features of plants (Homolová et al. 2013). We focused on ecophysiological traits and related them to forest drought responses as previous studies have shown that ecophysiological traits were closely linked to drought‐sensitive soil variables as well as different stages of forest development and local management (Schneider et al. 2017). Based on the functional diversity approach from ecophysiological traits initially suggested and applied to APEX imaging spectroscopy data by Schneider et al. (2017) and upscaled to Sentinel‐2 data by Helfenstein et al. (2022), we mapped three spectral indices at the canopy level.

We used a red‐edge chlorophyll index (CIre) to measure leaf chlorophyll content (CHL), a carotenoid/chlorophyll index (CCI) to measure leaf carotenoid/chlorophyll ratio (CCR), and a normalized difference infrared index (NDII) to measure leaf equivalent water thickness (EWT). All index maps were rescaled to 0–1.

CHL was obtained using CIre according to Clevers and Gitelson (2013) as

(1) $$\text{CIre}=\frac{\rho_{783}}{\rho_{704}}-1$$

where *ρ* stands for the top‐of‐canopy reflectance at a specific wavelength in nm. We used Sentinel‐2 bands B7 and B5. CIre from Sentinel‐2 correlated strongly with canopy CHL measured for field‐collected leaves and needles in a mixed mountain forest (Ali et al. 2020).

CHL affects photosynthesis, resource strategies (Croft et al. 2017), and growth potential by influencing how efficiently a plant can convert light into biomass. High chlorophyll content indicates a higher potential for photosynthesis and links to productivity.

CCI was developed for MODIS data to describe CCR and was successfully applied to Sentinel‐2 data (Helfenstein et al. 2022). CCI was calculated according to Gamon et al. (2016) as

(2) $$\mathrm{CCI}=\frac{\rho_{560}-\rho_{664}}{\rho_{560}+\rho_{664}}$$

We used Sentinel‐2 bands B3 and B4 for this calculation.

CCR reflects strategies for protecting photosynthesis against excess light input, which can impact growth rates and survival under stress.

The Normalized Difference Infrared Index (NDII) was used for the retrieval of EWT. We used the narrow infrared bands B8A and B11 (Helfenstein et al. 2022) and calculated the NDII according to Hardisky (Hardisky, Klemas, and Smart 1983).

(3) $$\text{NDII}=\frac{\rho_{865}-\rho_{1614}}{\rho_{865}+\rho_{1614}}$$

EWT is influenced by the structure (e.g., in broadleaf vs. needle trees), functions, and age of leaves and is affected by water availability, resource use, and conservation strategies.

We selected indices related to ecophysiological canopy traits for both ecological and technical reasons. Ecophysiological canopy traits are linked to growth, reproduction, and survival (Violle et al. 2007; Liu et al. 2016). They can be related to the leaf economic spectrum (Díaz et al. 2016) through their influence on photosynthetic efficiency, photoprotection, and water use strategies. The combination of the three selected traits, chlorophyll, carotenoid/chlorophyll ratio, and water content, describes canopy productivity and the tradeoff between productivity and protection that is important for response to disturbance. For example, young, productive forests and trees typically show high CHL and EWT as they invest energy in growth (Finegan 1984; Day, Greenwood, and White 2001; Acker et al. 2002). They tend to be less robust to stress, with most energy allocated to growth rather than defense, health, and reproduction (Loehle 1988; Obeso 2002). Carotenoids serve as antioxidants, ensuring the protection of leaves against high radiation and increased temperatures (Demmig‐Adams and Adams 1996; Lichtenthaler 2007).

We tried to avoid potential correlations among the selected indices, as a high correlation of the traits in the n‐dimensional trait space can negatively affect the calculation of the diversity metrics (Schneider et al. 2017; Blonder et al. 2018). All three selected indices show a low correlation with each other, which is a technical prerequisite for the functional diversity calculation. Furthermore, as seen in the selection of bands, the chosen indices do not overlap along the electromagnetic spectrum, providing independent measurements while using a broad variety of the spectral information provided by Sentinel‐2. In addition, it was discussed that the three traits investigated represent different plant responses to water limitation, each acting at a different temporal scale (e.g., Damm et al. 2018).

### 2.5 Functional Diversity Measures and Maps

**FIGURE 2:**
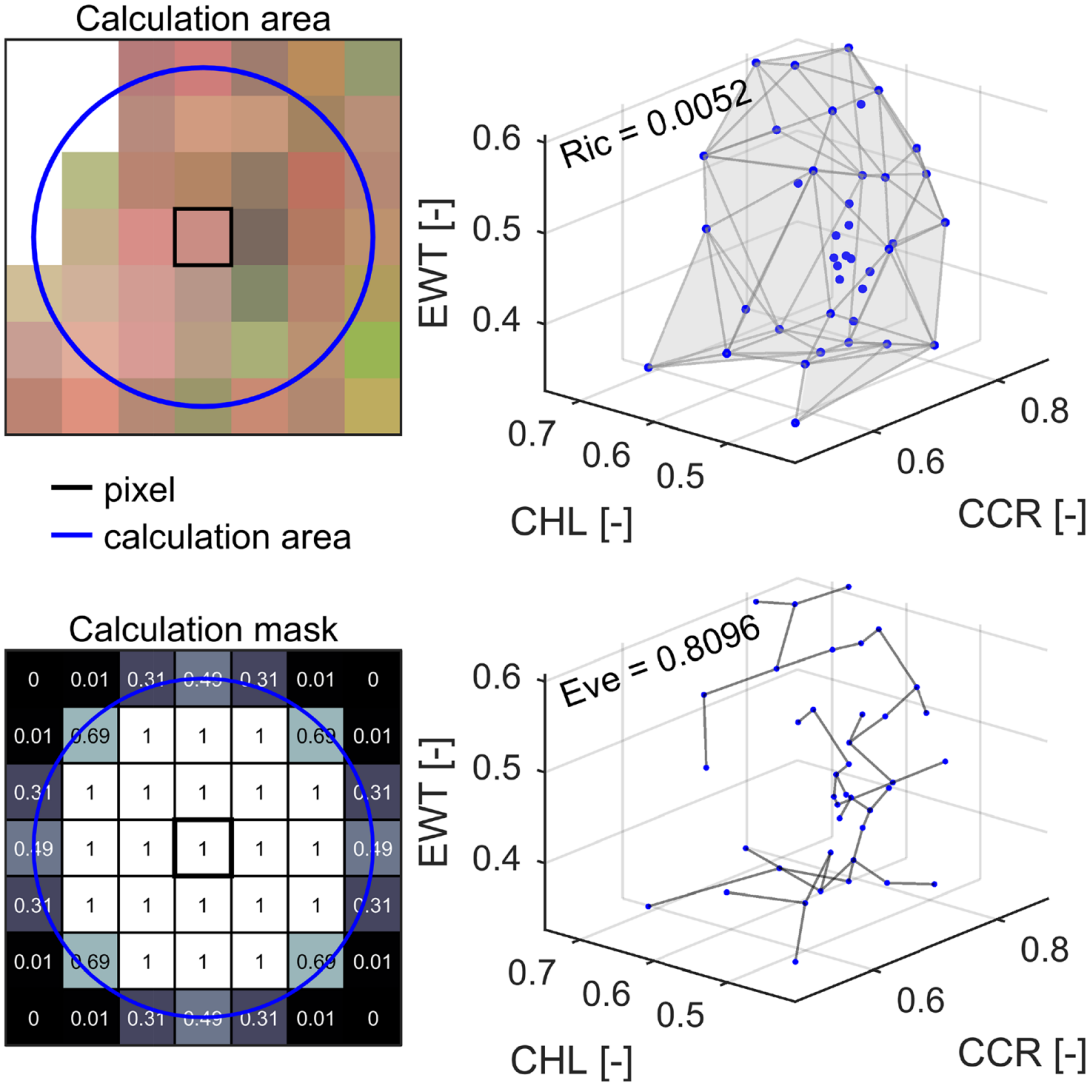
Calculation of diversity metrics from traits within the calculation area (top left). Shown is an example translation of the 60‐m radius (blue circle) neighborhood area to a mask for the calculation (bottom left). The numbers indicate the weighting of each pixel in calculating the value of the center pixel. Concepts of diversity metrics (right) in three‐dimensional trait space. Richness (Ric) (top right) and evenness (Eve) (bottom right). The traits considered include chlorophyll content (CHL), carotenoid/chlorophyll ratio (CCR), and equivalent water thickness (EWT).

Trait‐based functional diversity measures were derived from the per‐pixel trait values using a moving window approach with a circular calculation mask. Based on a previous scaling analysis, we used a three‐pixel calculation radius (i.e., 60 m when working with 20‐m pixels) to represent the patchy forest in the study area with a minimized risk of calculation‐based edge effects (Helfenstein et al. 2022). Figure 2 shows the calculation and the resulting mask for the moving window. A 60 m radius results in a calculation area of 28.3 pixels or 1.131 ha (Supporting Information: Section S2 showing the outcome of a multiscale analysis). The calculation radius of 60 m has previously been used to represent variation on the ecosystem to landscape scale (Zheng et al. 2023).

We used two metrics of trait‐based functional diversity (Figure 2), namely functional richness and evenness, calculated in the three‐dimensional space of the selected traits (Mammola et al. 2021; Villéger, Mason, and Mouillot 2008). These represent distinct dimensions of diversity (Rossi et al. 2020) and allow testing of the two hypotheses stated at the end of our Introduction section. Our functional richness and evenness measures were independent of each other (coefficient of determination of *r* ^2^ = 0.001 in the study area).

We calculated functional richness using concave hulls based on α‐shapes around the data points to reduce sensitivity to outliers compared to convex hulls (Gruson 2020). We complemented this with functional evenness to represent the regularity dimension of the data in the trait space. Evenness was calculated based on the minimum spanning tree (MST) using Euclidean distances between all points in trait space (Schneider et al. 2017; Villéger, Mason, and Mouillot 2008). Functional evenness measures the regularity of the shape of the occupied trait space from the length of the branches in the MST and the evenness in their abundance. The index is derived by normalizing edge weights in the MST and accumulating a sum of minimum partial weighted evenness across vertices, normalized against theoretical minima (Villéger, Mason, and Mouillot 2008).

### 2.6 Drought Response Maps

Our approach to quantifying drought response in forests was based on Sturm et al. (2022). We calculated the normalized difference water index (NDWI) after Gao (1996) using the reflectance in bands B8 NIR and B11 SWIR1 as

(4) $$\text{NDWI}=\frac{\rho_{833}-\rho_{1614}}{\rho_{833}+\rho_{1614}}$$

Change in NDWI has been shown to be sensitive to water stress (Marusig et al. 2020). The August NDWI values were calculated for each year from 2017 to 2020 by taking the median NDWI value from the images in Table 1.

**FIGURE 3:**
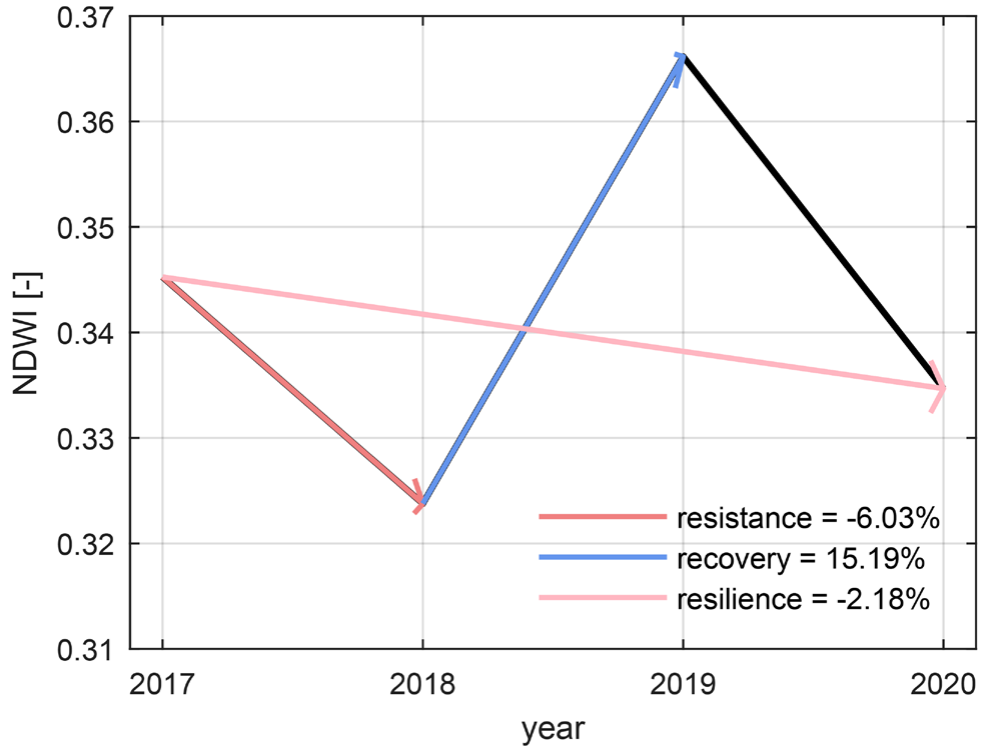
Trend of the mean normalized difference water index (NDWI) in the study area between 2017 and 2020. The numbers in the legend represent the mean percent changes for the three drought response measures (change 2017 to 2018 resistance, change 2018 to 2019 recovery, and change 2017 to 2020 resilience) across the entire study area in northern Switzerland.

We assessed the response of forests to the 2018 drought year by comparing the relative pixel‐wise percentual change between base NDWI conditions in August 2017 and conditions during the drought (2018) or post‐drought (2019, 2020) years (Figure 3). Similar to van Moorsel et al. (2021), we defined resistance as the NDWI change ratio between 2017 and 2018 [(NDWI_2018_ − NDWI_2017_)/NDWI_2017_] to assess immediate changes happening during the event, and we defined recovery as the change ratio between 2018 and 2019 [(NDWI_2019_ − NDWI_2018_)/NDWI_2018_] to assess post‐drought changes. Additionally, we defined resilience as the change ratio between 2017 and 2020 [(NDWI_2020_ − NDWI_2017_)/NDWI_2017_]. We used the second (2020) rather than the first post‐drought year (2019) to avoid a linear combination of resilience and recovery (Gazol and Camarero 2016) and to partially account for potential time‐lagged drought response effects, which might be visible in additional physiological changes or secondary drought effects like bark beetle outbreaks (Stroheker, Forster, and Queloz 2020).

NDII and NDWI are two different indices related to canopy water content; however, they share bands in their definition. Both the diversity measures and the drought response measures were mapped using satellite data from the same platform, which might introduce spurious correlations. However, we designed the experiment to minimize potential effects. We differentiated between NDWI and NDII using the NIR band 8 for NDWI and the overlapping band 8A for NDII and used Sentinel‐2 data at different times of measurement (Table 1). For further analysis, we mapped functional diversity using the spatial distribution of EWT from NDII combined with two other ecophysiological traits to describe diversity (spatial dimension) and pixel‐based annual relative change using NDWI to describe drought response (temporal dimension). Therefore, while we used water content values for diversity and drought response as part of their calculation (spatial distribution and relative annual change), diversity and drought response are based on independent observations.

### 2.7 Separate Analysis of Drought Responses to Functional Richness and Evenness

Small and isolated patches of forest were excluded from the calculation following Helfenstein et al. (2022) because their functional diversity measures were affected by edges. This step removed 14.35 km^2^ or 1.57% of the forest area. We then applied binning to the diversity data to examine the spatial distribution of diversity values. The binning process over the whole study area reduces potential autocorrelation effects because adjacent pixels with similar values will be combined, and pixels with different values will be separated. We formed 1000 bins of equal range within diversity metrics and averaged drought response values within each bin. Before binning, we conducted image preprocessing by rescaling to a range of 0–1, with the lowest 0.1% set to 0 and the highest 0.1% set to 1. This approach avoided generating empty or small bins that could introduce bias to our subsequent analysis. After the binning process, we excluded bins that contained less than 1% of the maximum pixel number per bin. Functional richness was divided into 823 bins with values ranging between 0 and 0.261. Functional evenness was divided into 861 bins with values ranging between 0.6974 and 0.8698. Results without exclusions of bins were very similar and presented in Figure S9. We then used the binned values to investigate the drought responses to functional richness and evenness in separate linear regression models. The numbers of pixels per bin were used as weights.

### 2.8 Combined Analysis of Drought Responses to Functional Richness and Evenness

We employed linear models to examine the relationships between drought response (resistance, recovery, and resilience) and the two functional diversity measures, treating the latter as explanatory variables. For this combined analysis, we discretized the explanatory variables into 20 bins and incorporated 21 geographic subregions to account for geographical variation. This resulted in a dataset comprising 8400 strata (20 richness bins × 20 evenness bins × 21 subregions) (Figure 5). Note that this procedure ensures that the three variables functional richness, functional evenness, and subregion are more or less orthogonal to each other, with correlations among them only due to the potential occurrence of empty bins.

We directly analyzed the mean NDWI change for each bin while considering forested pixels per bin (*N*) as a weighting variable to emphasize bins with more data. We used the linear models to obtain percentages of total sum of squares (SS) for the different explanatory terms and their interactions in the model (increments of multiple *r* ^2^ ×100). In all models, we used the drought response measures as continuous variables, functional diversity measures binned into 20 bins, and subregion (REG) as a categorical variable with 21 levels representing the spatial variation. We iteratively refined the models, fitting subregion, functional diversity measures, and interactions (*x*).

Richness (ric) showed a linear relationship with resilience (rsl) and a non‐linear relationship with resistance (rst) and recovery (rcv), necessitating a logarithmic transformation (logric) in the latter case. Similarly, because evenness (eve) showed a hump‐shaped relationship in all models, we fitted a polynomial (eve^2^). Non‐significant explanatory terms (*p* ≥ 0.05) or explanatory terms with SS < 1% were excluded from the models. We used the Akaike information criterion (AIC) and *r* ^2^ to determine the optimal model from linear, quadratic, and logarithmic regressions. AIC helps balance goodness of fit with model complexity, while *r* ^2^ helps assess how well each model describes the variance in the data. This procedure resulted in the following linear models using R notation (R Core Team 2024):

1. $\begin{array}{c} \mathrm{lm}(\text{terms}(\mathrm{rst}\sim \text{logric}+(\mathrm{eve}+{\mathrm{eve}}^{2})+\mathrm{REG}+\text{logric}x\mathrm{REG}\\ +\mathrm{eve}x\mathrm{REG}+{\mathrm{eve}}^{2}x\mathrm{REG},\text{keep}.\text{order}=T),\;\text{weight}=N) \end{array}$
2. $\begin{array}{c} \mathrm{lm}(\text{terms}(\mathrm{rcv}\sim \text{logric}+(\mathrm{eve}+{\mathrm{eve}}^{2})+\mathrm{REG}+\text{logric}x\mathrm{REG}\\ +\mathrm{eve}x\mathrm{REG}+{\mathrm{eve}}^{2}x\mathrm{REG},\text{keep}.\text{order}=T),\;\text{weight}=N) \end{array}$
3. $\begin{array}{c} \mathrm{lm}(\text{terms}(\mathrm{rsl}\sim \mathrm{ric}+\mathrm{eve}+\mathrm{REG}+\mathrm{ric}x\mathrm{REG}\\ +\mathrm{eve}x\mathrm{REG},\text{keep}.\text{order}=T),\;\text{weight}=N) \end{array}$

Here ric = richness, eve = evenness, logric = log(richness), eve^2^ = evenness squared, REG = subregion, *x* = interaction operator (indicating how the effect of one variable depends on another, for example, how diversity effects vary across subregions), and *N* = the number of forested pixels per bin.

We also tested the functional diversity effects using their interactions with subregions as error terms to obtain a more conservative *F*‐ratio (F2 in Tables S2–S4). Note that this corresponds to a data analysis using linear mixed models with the interactions as random terms (Nelder and Lane 1995; Schmid et al. 2017).

In the above analyses, functional richness effects are tested across subregions, with the interaction term testing for differences in functional richness effects between subregions. For Figure 5, richness effects corrected for subregions were calculated by fitting subregions first in the above linear models. For plotting the corrected data, we added the residuals from a linear model fit with subregions as an explanatory term to the overall mean.

## 3 Results

### 3.1 Biodiversity Data

**FIGURE 4:**
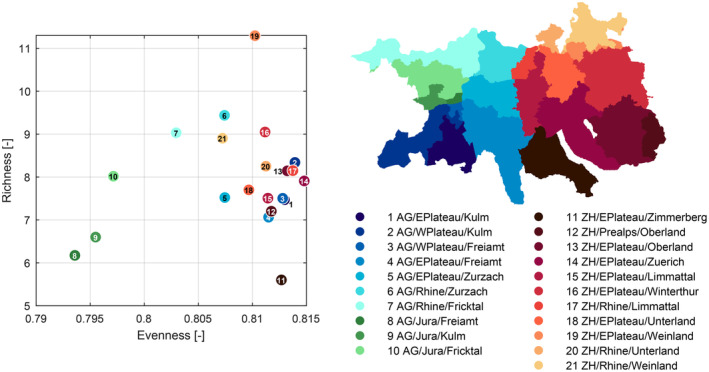
Average diversity of 21 subregions with the plot on the left showing their median richness and evenness. It is important to note that the variation within the regions is large, and the differences between regions are comparatively small (see Figure S6). The subregions shown on the right were obtained by grouping the forests of the study area according to the intersection of (1) canton (Aargau [AG] and Zurich [ZH]), (2) geographical regions (Central Plateau [Eastern and Western], Rhine plains, Jura, and Pre‐Alps), and (3) four, respectively seven, cantonal forest districts. Blue‐green colors represent canton AG, and red‐yellow colors represent canton ZH. The color gradients range from southern to northern regions within the cantons.

We calculated diversity maps based on the three canopy traits, chlorophyll content, carotenoid/chlorophyll ratio, and equivalent water thickness, as assessed from spectral reflectance indices (Figures S2 and S3). The three trait maps only showed weak linear correlation with each other, with coefficients of determination of *r* ^2^ = 0.215 for CHL and CCR, *r* ^2^ = 0.185 for CHL and EWT, and *r* ^2^ = 0.055 for CCR and EWT (Figure S2). The scatterplot in Figure 4 shows the distribution of richness and evenness among the 21 subregions. The northern subregions of the study area had higher richness than the southern regions. Richness was highest in subregions of the Rhine plain (6, 7, 17, 20, 21), and the lowlands of the Swiss Plateau (2, 13, 16, 19). Subregions of lower richness were found toward the south (4, 11, 12). Regarding evenness, subregions in the south (1, 3) and southeast (11, 12, 14) showed high values, with the northern subregions (5–7, 21) showing lower values. The three Jura subregions (8–10), with low richness and evenness values, differ from the rest of the study region. Although these differences in mean richness and evenness between subregions were significant, variation in richness and evenness within subregions was also large (Figure S6).

### 3.2 Drought Response Metrics

We derived drought response values across the study area based on the Normalized Difference Water Index (NDWI) data. The entire study area was strongly affected by the drought in 2018, which was visible in a reduction of the NDWI from 2017 to 2018 (see Figure 3). From 2018 to 2019, the forest in the study area showed an increase in NDWI, followed by a second but weaker decrease in 2020 to a level slightly lower than in 2017 but higher than in 2018. Low resistance values (< −7.5% in 38.6% of the area, see Table S1) occurred in the northern lowlands (Figure S5, top). Most of the forested area (73.5%) showed a > 7.5% increase in NDWI from 2018 to 2019 (Figure S5, middle). Resilience values < −7.5% occurred across 28.5% of the area, especially in the southern regions (Figure S5, bottom). We validated the 2020 resilience maps using a classified dataset based on visual interpretation of aerial images (see Supporting Information: Section S1). Visually damaged areas showed a significantly different RS‐derived drought response than visually non‐damaged areas (Figure S12).

### 3.3 Relationships Between Diversity Metrics and Drought Responses

We first analyzed the relationship between drought resistance, recovery, or resilience and diversity metrics separately for functional richness and evenness, grouping these measurements into 1000 bins each. The correlation between the satellite‐derived maps before and after binning is illustrated in Figure S7. Using AIC and *r* ^2^ to determine the optimal model from linear, quadratic, and logarithmic regressions, we found approximately logarithmic relationships between resistance or recovery and functional richness, while relationships between resilience and functional richness and evenness were approximately linear (Figure S8). Resistance increased, and recovery decreased with richness at low values of richness, and then tempered off, whereas resilience generally increased with richness, but with a plateau at intermediate richness levels (Figure S8, top row). Resistance and recovery also increased and decreased, respectively, with evenness at low values of evenness, but at high values, the relationship reversed; resilience generally decreased with increasing evenness (Figure S8, bottom row).

**FIGURE 5:**
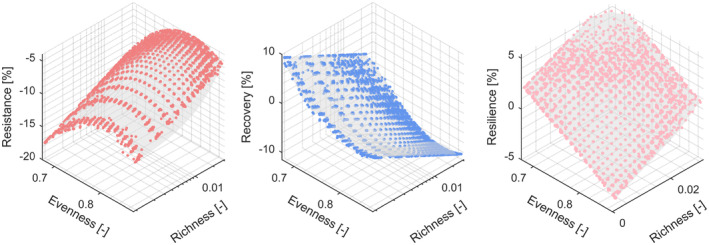
Subregion‐corrected drought resistance, recovery, and resilience (left to right) in relation to functional richness and evenness. These data were binned into 20 bins along richness and evenness and into the 21 subregions, resulting in 8400 bins. We then fitted subregion to correct for subregion differences and finally related the thus corrected drought responses to functional richness and evenness using multiple regression (as described in Section 2). Resistance and resilience increased with richness. Resistance showed a hump‐backed relationship with evenness, while resilience decreased with evenness.

We then analyzed the relationships between drought responses and functional richness and evenness in combined models, aggregating data using 20 bins each for the two diversity metrics crossed with the 21 subregions, yielding a data table with 20 × 20 × 21 = 8400 rows. All bins showed a reduction in NDWI in 2018 (i.e., no bins were fully resistant) and an increase in 2019 (i.e., positive recovery) (Figure 5).

**FIGURE 6:**
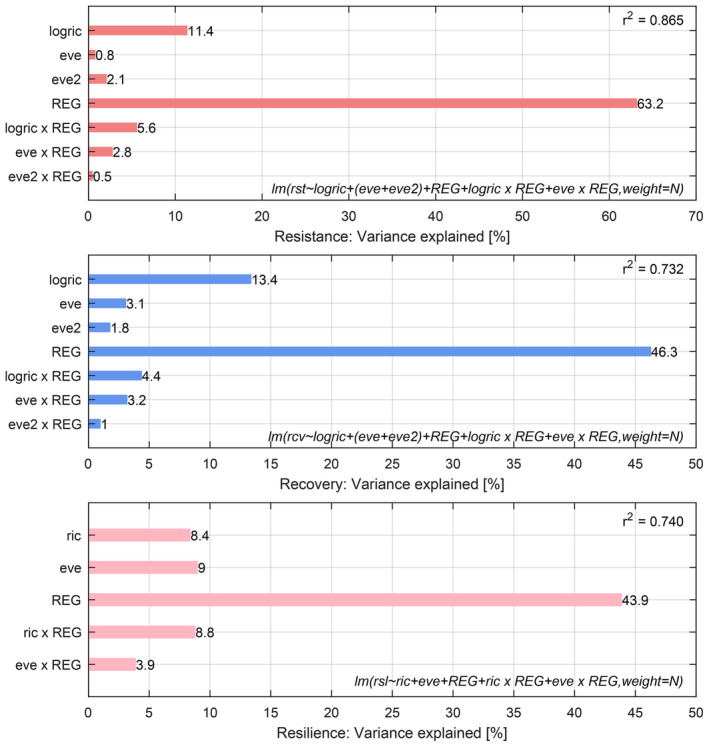
Variance explained by the linear model combining the influence of the diversity metrics richness and evenness on resistance (change in NDWI during the drought 2017–2018, Table S2), recovery (change in NDWI after the drought 2018–2019, Table S3), and resilience (change in NDWI after the full 2‐year observation period 2017–2020, Table S4). The bars from top to bottom in each panel are the contributions to the *r* ^2^ values of linear richness (ric), log‐transformed richness (logric), evenness (eve), evenness squared (eve^2^), the 21 subregions (REG), and interactions of the diversity metrics and subregions (ric × REG, eve × REG). Note that all contributions are significantly larger than zero. The formulae for the fitted linear models are listed in R notation, with *N* representing the number of pixels per bin.

The best‐fitting linear models showed the primary role of functional richness as a predictor for both resistance and recovery, yet similar roles for functional richness and evenness as predictors for resilience (Figure 6; Tables S2–S4). The overall relationships between drought responses and functional richness or evenness were similar when fitted before or after, that is, corrected for, differences between subregions (the latter was used to display the results in Figure 5). When we compared the BEF relationships between subregions, significant differences were detected, but these were small compared with the average overall relationship (Figure S10; Tables S2–S4). That is, if mean squares for the diversity metrics were divided by the mean squares for the corresponding interactions with region, the resulting *F*‐ratios were all significant (Tables S2–S4). The regional slopes of resilience as a function of richness and evenness are shown in Figure S11.

## 4 Discussion

Our results show an overall positive relationship between RS‐derived functional diversity in leaf physiological traits and RS‐measured drought responses of forests across an area of 3133 km^2^ in northern Switzerland, assessed in a scalable approach from satellite remote sensing. Based on results from plot‐scale BEF experiments in grassland and forest ecosystems (Schmid et al. 2009), we hypothesized that more diverse forests should have suffered less from an extreme drought event occurring in 2018 across central Europe. We hypothesized a positive relationship between drought response and functional richness and a hump‐shaped relationship between drought response and functional evenness, the latter due to dominance effects being correlated with less than maximum evenness. Both hypotheses were broadly supported by our satellite‐derived dataset. Furthermore, richness effects were generally stronger than evenness effects, as expected from BEF experiments.

Similar to BEF experiments (Isbell et al. 2015), drought resistance in our observational study increased linearly with the logarithm of functional richness across 18 subregions, with only three subregions showing non‐positive relationships. Recovery was negatively related to the logarithm of richness, but resilience overall increased linearly with untransformed richness, although five out of the 21 subregions showed negative responses. The direct link of functional richness with EF is in agreement with other studies; for example, it was found that structural complexity, rather than species diversity alone, explains positive tree richness–productivity relationships in BEF experiments (Ray et al. 2023). Furthermore, recent studies point out the importance of functional traits for understanding forest drought responses, as observed response patterns to drought vary widely among studied species (Pardos et al. 2021). High functional richness likely increases the probability for complementary drought reactions among tree species, thus leading to higher resistance and resilience at the level of entire forest stands. In addition, with higher functional richness, it is more likely that a forest stand includes tree species that can contribute strongly to the drought response of the stand and that this will be reflected in uneven abundance distributions among species and thus reduced trait evenness. These two effects resemble complementarity and selection (dominance) effects obtained in additive‐partitioning schemes for net biodiversity effects in BEF experiments (Isbell et al. 2018; Loreau et al. 2001).

The hump‐shaped or negative relationships of drought resistance or resilience, respectively, with functional evenness indicated that a certain level of dominance was beneficial for forest stands of a given trait richness under drought. In our study, richness and evenness effects were uncorrelated because functional evenness was calculated as regularity within a given hypervolume reflecting functional richness. Thus, functional evenness could not account for differences in functional richness and vice versa (see Figures S4 and S7). Furthermore, functional richness and evenness effects were additive; that is, there were no interactive effects of the two on drought responses. Thus, the highest drought resistance was observed in forests with high functional richness and intermediate levels of functional evenness, and the highest drought resilience was observed in forests with high functional richness and low functional evenness (see Figure 5). This suggests that a combination of complementarity and dominance effects underpins the relationships of forest drought responses with trait‐based functional diversity in the studied temperate forests. Dominant species play a major role in the stability of dry grasslands (Wang et al. 2022), but how this is related to functional richness and evenness is unknown. A caveat that remains is that, in our study, functional evenness was measured before the extreme drought in 2018 and thus could not be a response to it. However, it is conceivable that for some forest stands, the earlier, less extreme drought events occurring in 2011 and 2015 (MeteoSchweiz 2020) had led to trait dominance of trees with more resistant and resilient drought responses. This could then have predisposed these stands to show more resistant and resilient responses to the extreme drought event in 2018.

Additionally, the Years 2017, 2019, and 2020 were also comparatively warm and dry, although they lacked the prolonged drought phases observed in 2018 (see Figure S1). While these years serve as a reference in this study, physiological responses might already have influenced observed resistance and resilience and limited recovery. Ideal [i.e., closer to average conditions observed between 1961 and 1990 (MeteoSchweiz 2019)] reference years that might have occurred before the Sentinel‐2 satellite record started (Immitzer, Vuolo, and Atzberger 2016) would provide a potentially better pre‐drought baseline. However, the use of recent and thus warmer and/or drier reference years in our study should not have diminished the overall robustness of our results because ideal reference years would likely have led to even larger estimates of forest responses to drought stress. Furthermore, given the increased frequency of dry and warm conditions in recent years across central Europe (Jacob et al. 2014), our reference years might be considered the new “normal.” Because we focused on the analysis of change in canopy water content, we here did not isolate secondary drought effects (e.g., dieback and pests) from direct drought damage. This would be an important consideration for future studies incorporating longer temporal ranges, including post‐2020 data, to better isolate secondary drought effects and improve reference year selection. This will introduce additional complexity when attributing damage to a specific drought event.

In contrast to other ecosystem functions such as primary productivity and resistance to other disturbances such as pest outbreaks (Jactel, Moreira, and Castagneyrol 2020), evidence about the impact of mixed forests on drought damage so far has been largely lacking (Bauhus et al. 2017), although evidence is available from grassland biodiversity experiments (Pfisterer and Schmid 2002; Isbell et al. 2015). Challenges in understanding the biodiversity–drought response relationships may arise from the large scale and low selectivity at which droughts occur, driven by broad climate impacts across extensive forested areas (Jactel et al. 2017).

We observed a clear dependence between resistance and recovery when stratified for different diversity metrics, that is, bins with lower resistance in 2018 showed increased recovery in 2019. This observation indicates a compensatory response and is consistent with previous findings by Sturm et al. (2022), who speculated that reduced competition following tree die‐back in 2018 may have caused it. Resistance and recovery have also been shown to be negatively related in previous experimental and observational studies (Gazol et al. 2017). This negative correlation dampens variation in resilience, yet similarities between the resistance and resilience responses to the drought in our studies indicate that the recovery responses could only partly compensate for low resistance. Similar observations have previously been made in diverse forests (Anderegg et al. 2018; Gazol and Camarero 2016; Pardos et al. 2021; Sturm et al. 2022) and suggest that ecosystem stability may generally be more strongly related to resistance than to recovery, with the latter being a “passive partial compensation” of the former. Therefore, we suggest focusing on resistance for predicting stability responses to extreme events such as the 2018 drought across central Europe. To disentangle resilience from this compensatory effect, we added an additional post‐drought year to assess resilience in addition to the post‐drought year 2019 used by Sturm et al. (2022).

High biodiversity is suggested to promote forest resilience to climate change (Messier et al. 2019), although field‐based evidence is still scarce, especially regarding trait‐based functional diversity (Mori, Lertzman, and Gustafsson 2017). However, studies suggest that intraspecific variation in functional traits plays a crucial role in regulating drought resilience in forests (de Andrés et al. 2021). Here, we present an approach to link trait‐based forest drought responses to functional diversity at landscape scales using satellite data in a scalable manner. This approach is promising for assessing and predicting forest drought responses in other regions and over time, as the spectral indices used to calculate functional diversity can be measured in near real‐time. The thus obtained functional diversity measures for 20 m pixels may be only indirectly related to field‐based measures of diversity in leaf ecophysiological traits (Schneider et al. 2014). The 20 m resolution of the Sentinel‐2 imagery leads to an averaging of multiple trees' intra‐individual and inter‐individual trait values within each pixel (see Figure S12), leading to an underestimation of the trait variance and resulting functional diversity. Previous research using high‐resolution airborne spectroscopy data in the research area has shown that coarser resolutions reduce observed trait variance but still capture meaningful patterns of functional diversity relevant to ecosystem processes (Helfenstein et al. 2022).

Furthermore, our results suggest that these trait measures derived from Sentinel‐2 imagery pick up relevant components of biodiversity related to forest drought responses. Forests with greater functional diversity, as assessed from Sentinel‐2 imagery, are better protected against drought than forests with lower RS‐derived functional diversity. The mechanisms underlying this relationship need further investigation. Functional diversity in leaf ecophysiological traits might also link to drought‐sensitive soil variables (Schneider et al. 2017). The stabilizing effect of this functional diversity might emerge from asynchronous drought responses of functional types of species or individuals (Schnabel et al. 2021). Leaf and canopy ecophysiological diversity might also link to functional diversity of plant hydraulic traits, such as stem water potential, which were found to explain drought‐induced tree mortality (Anderegg et al. 2018). Our understanding of these mechanisms will benefit from the integration of both field‐based and RS approaches to obtain a comprehensive understanding of how trait‐based diversity explains or predicts forest resilience across various contexts.

Trait‐based functional diversity of forest canopies, as derived from satellite data, differs from typical field‐based functional diversity measures calculated from species means or individual‐tree values. There is a need for a systematic evaluation of the links between RS‐derived and field‐based functional diversity measures. Extensive trait sampling within a pixel area would be important to represent the community level as measured by satellites. Validation datasets optimized to capture the spatial, temporal, and species representativeness of satellite data would enable better validation of RS‐based trait estimates (Cavender‐Bares et al. 2022). Furthermore, additional work is needed to fill the information gap between leaf measurements and satellite data. Trait measurements using close‐range RS (e.g., from drones or airborne platforms) might be helpful, as well as the upscaling of leaf‐level optical properties to canopy spectra using radiative transfer models (RTMs) (Schneider et al. 2014). Still, the availability of global satellite data indicates that the method presented here can be applied to other temperate forest regions, provided that temporal and spatial coverage are sufficient. RS‐derived functional diversity measures hold the promise that they can be obtained without the need to distinguish species and individuals and could thus enable generalization across the forest ecosystems of the world and their highly diverse species compositions (Kunstler et al. 2016). The impact of droughts varies greatly in biomes of different climatic regions (Liu et al. 2021). Using the RS‐derived functional diversity measures and drought responses introduced here, the stability of ecosystems to other disturbances such as pathogen outbreaks or fires could also be investigated (Jetz et al. 2016). Advances in approaches to analyze satellite RS products to map forest disturbances at large scales and analyze patterns in disturbance size, frequency, and severity will support this work (Senf and Seidl 2021). Forest masks needed for this approach can either be derived from governmental maps, as used here, or from LiDAR‐derived vegetation height (Helfenstein et al. 2022). However, availability is usually geographically limited. Standardized inventories or frameworks for combining Sentinel‐2 data and 3D information could support the upscaling of the approach to global applications (Valbuena et al. 2020).

The adaptability of the presented approach highlights the potential for translation to landscapes worldwide. Switzerland's small‐scale heterogeneity and availability of detailed environmental data allowed for the use of finely resolved subregions and facilitated the analysis of the variability of the observed patterns on smaller scales. Similar patterns over larger geographic extents may require alternative subregion definitions, such as bioclimatic zones, ecoregions, or clustering methods, to effectively capture spatial variation in functional diversity and responses.

Multispectral sensors like Sentinel‐2 offer limited spectral bands compared with sensors of high spectral resolution, reducing the dimensions available to derive vegetation properties. The three traits derived from Sentinel‐2 imagery in our study show a link with drought response, but a more diverse set of traits could provide a more comprehensive understanding. Imaging spectroscopy expands possibilities for deriving vegetation traits and drought‐sensitive indicators, spanning from specific leaf ecophysiological traits to mapping functional or phylogenetic diversity (Meireles et al. 2020). Recent and upcoming spaceborne imaging spectrometers will advance spaceborne diversity and forest health monitoring (Cawse‐Nicholson et al. 2021). Furthermore, various diversity measures for remotely sensed data were presented in recent years, such as Rao's Q (Botta‐Dukát 2005; Pangtey et al. 2023; Rocchini, Marcantonio, and Ricotta 2017). We used a complementary set of diversity metrics, richness, and evenness to provide clear insights into how these distinct facets of diversity relate to drought responses. A single measure incorporating aspects of these metrics may be useful for a concise indicator of diversity, for example, Petchey and Gaston's functional diversity measure based on branch lengths in a dendrogram calculated from species trait values (Petchey and Gaston 2002). However, this measure could not be calculated in the absence of species identification. Furthermore, Rao's Q as a measure based on mean distances does not account for important richness effects of functional diversity. For these reasons, we here decided not to complement the presented metrics with further measures.

There is a need to study EF within the global biodiversity monitoring framework using satellite RS (Pettorelli et al. 2018). Existing field‐based datasets show geographic and temporal biases, mainly focusing on temperate ecosystems (Proença et al. 2017). Our scalable approach builds toward assessing large‐scale BEF relationships from satellite data, independently of the study area and over time. A major advantage of high‐resolution public satellite data is repeated and standardized information enabling the monitoring of BEF relationships. The relationships between RS‐derived functional diversity measures and forest drought responses assessed in the present paper might change over time or depending on the season the drought takes place. Monitoring these relationships using satellite data can reveal valuable information for adaptive management.

Insights presented here advance large‐scale assessments of the stability and resilience of non‐experimental ecosystems using satellites toward global monitoring of the impacts of biodiversity on EF. Our results indicate that trait‐based functional diversity at the canopy level supports forest responses to drought regardless of other stand characteristics and environmental context within a relatively homogenous region on the Swiss plateau. Increasing drought resistance positively relates to forest functional richness, while the observed hump‐shaped relationship of drought resistance with functional evenness suggests an optimum diversity in terms of functional evenness. Increasing drought resilience positively relates to functional richness and negatively relates to functional evenness. Our work explores and confirms the link between trait‐based functional diversity and forest drought response assessed using satellite data, contributes to understanding climate change impacts on forests, and provides the basis for further research on landscape‐scale BEF relationships. Derived insights contribute to establishing large‐scale assessment and long‐term monitoring of forest diversity and BEF using satellite data.

## Author Contributions

**Meredith C. Schuman:** project administration, resources, writing – review and editing. **Felix Morsdorf:** project administration, writing – review and editing. **Alexander Damm:** resources, writing – review and editing. **Joan T. Sturm:** conceptualization, data curation, methodology, visualization, writing – original draft. **Bernhard Schmid:** conceptualization, methodology, writing – original draft. **Isabelle S. Helfenstein:** conceptualization, data curation, formal analysis, investigation, methodology, validation, visualization, writing – original draft.

## Conflicts of Interest

The authors declare no conflicts of interest.

## Supporting information


Data S1.

## Data Availability

The data that support the findings of this study are openly available in Zenodo at https://doi.org/10.5281/zenodo.14678089 and in the GitHub repository https://github.com/ihelfens/RS‐TraitDiv‐DroughtResp. Sentinel‐2 data was obtained from the Copernicus Climate Change Service (C3S) Climate Data Store (CDS) at https://dataspace.copernicus.eu/explore‐data/data‐collections/sentinel‐data/sentinel‐2. The cantonal boundaries used in Figure 1 are available at https://opendata.swiss/en/dataset/swissboundaries3d. The forest area datasets were provided by the cantons Aargau and Zurich and are available at https://opendata.swiss/en/dataset/waldareal2 and https://www.geolion.zh.ch/geodatenservice/show?nbid=1257 (v1.1.0). The biogeographic regions of Switzerland are accessible at https://opendata.swiss/en/dataset/biogeographische‐regionen‐der‐schweiz‐ch. The forestry district data is available at https://opendata.swiss/en/dataset/76‐ag‐forstkreise‐und‐forstreviere and https://geolion.zh.ch/geodatensatz/show?giszhnr=226. The aerial data used for validation was provided by the canton Zurich and are available via the geolion data catalog at https://geolion.zh.ch/geodatensatz/show?gdsid=493 and https://geolion.zh.ch/geodatensatz/show?gdsid=527.
