# Supplementary material for: Satellite Observations Reveal a Positive Relationship Between Trait‐Based Diversity and Drought Response in Temperate Forests
Source: Glob Chang Biol. 2025 Feb 3;31(2):e70059. doi: 10.1111/gcb.70059 (PMC11789211; doi:10.1111/gcb.70059)
Supplement: Supplementary file 1 — Data S1. [file GCB-31-e70059-s001.pdf]

Supplementary Material: Satellite observations reveal a positive relationship between trait-based diversity and drought response in temperate forests

Corresponding author: Isabelle S. Helfenstein. Email address: isabelle.helfenstein@geo.uzh.ch

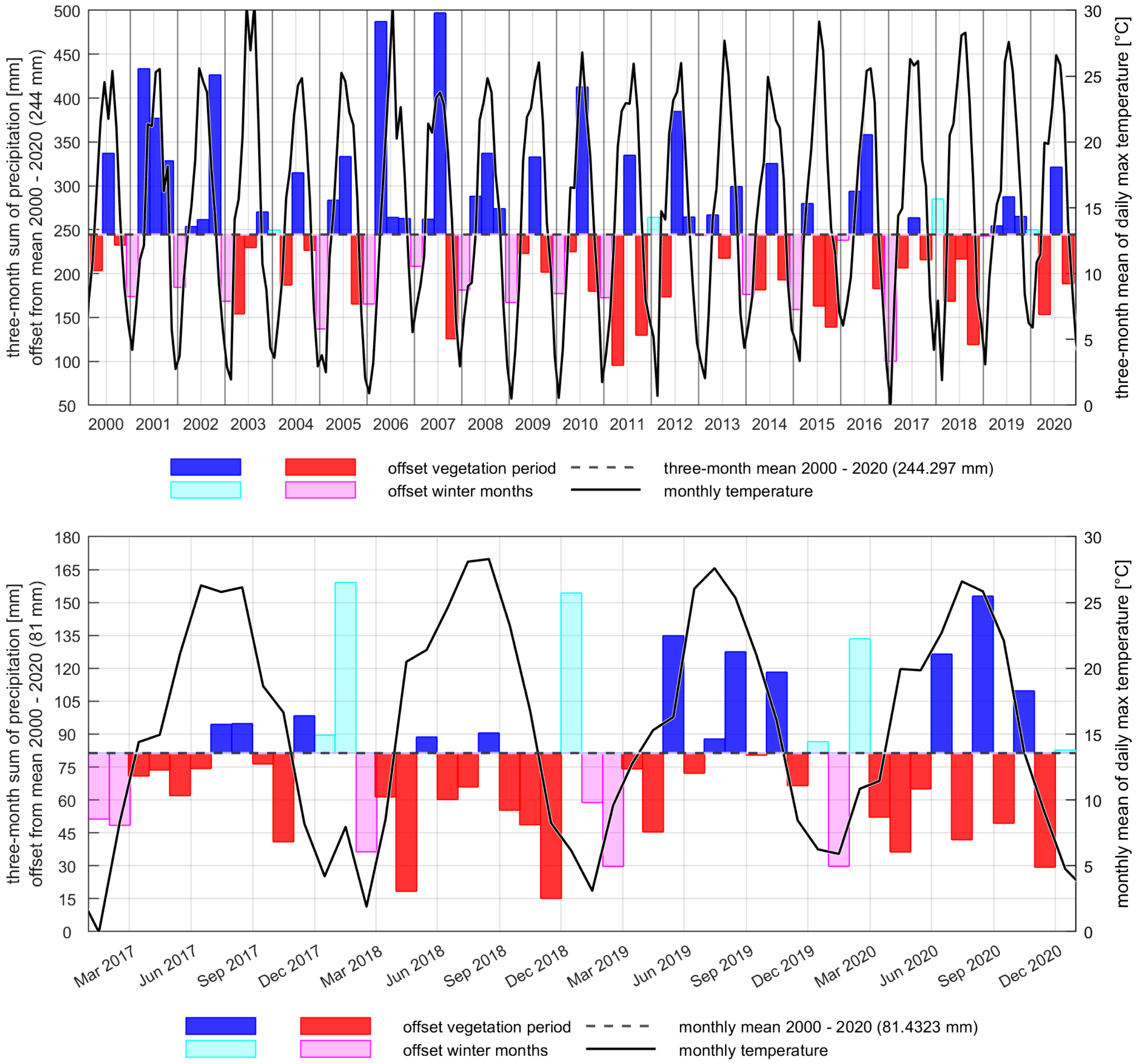

Figure S1: Sum of precipitation and mean daily maximum temperature combined (using mean calculation) for two weather stations, Kloten in the canton Zurich and Buchs/Aarau in the canton Aargau. Top: Three-month sum of precipitation offsets respective to the twenty-year three-month precipitation mean (dashed) and monthly mean of daily maximum temperature (solid). Blue bars show a positive offset, and red colors a negative offset, indicating wet and dry periods. In winter months (December, January, February), precipitation is indicated in light colors to accentuate the vegetation period (March-November). Bottom: Detailed view of the monthly sum of precipitation and mean of daily maximum temperature (solid) for the years 2017 – 2020. Blue and pink bars show offsets from the 2000 – 2020 monthly mean (dashed). All four years of interest were comparatively warm and dry, with 2018 showing the highest maximum temperature of the four and frequent as well as large negative offsets during the vegetation period, especially compared to 2019 and 2020. The data were provided by the Federal Office of Meteorology and Climatology MeteoSwiss.

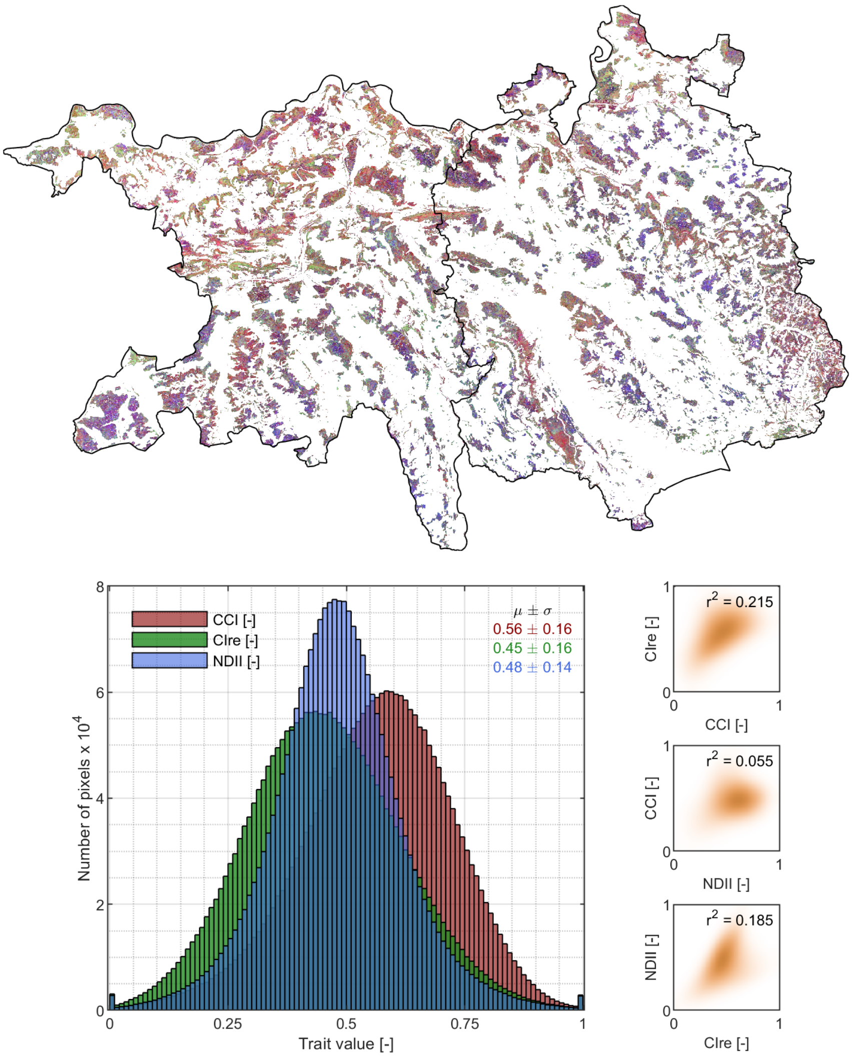

Figure S2: Top: calculated indices CIre (green), CCI (red), and NDII (blue) as proxies for the physiological traits CHL, CCR, and EWT at the research site and normalized between 0 and 1. Bottom left: histogram of physiological traits, including means and standard deviations. Bottom right: coefficient of determination of vegetation indices. CIre and CCI show the highest coefficient of determination with r^2 = 0.215, followed by CIre and NDII with r^2 = 0.185 and CCI and NDII with r^2 = 0.055. The individual trait maps are illustrated in Figure S3.

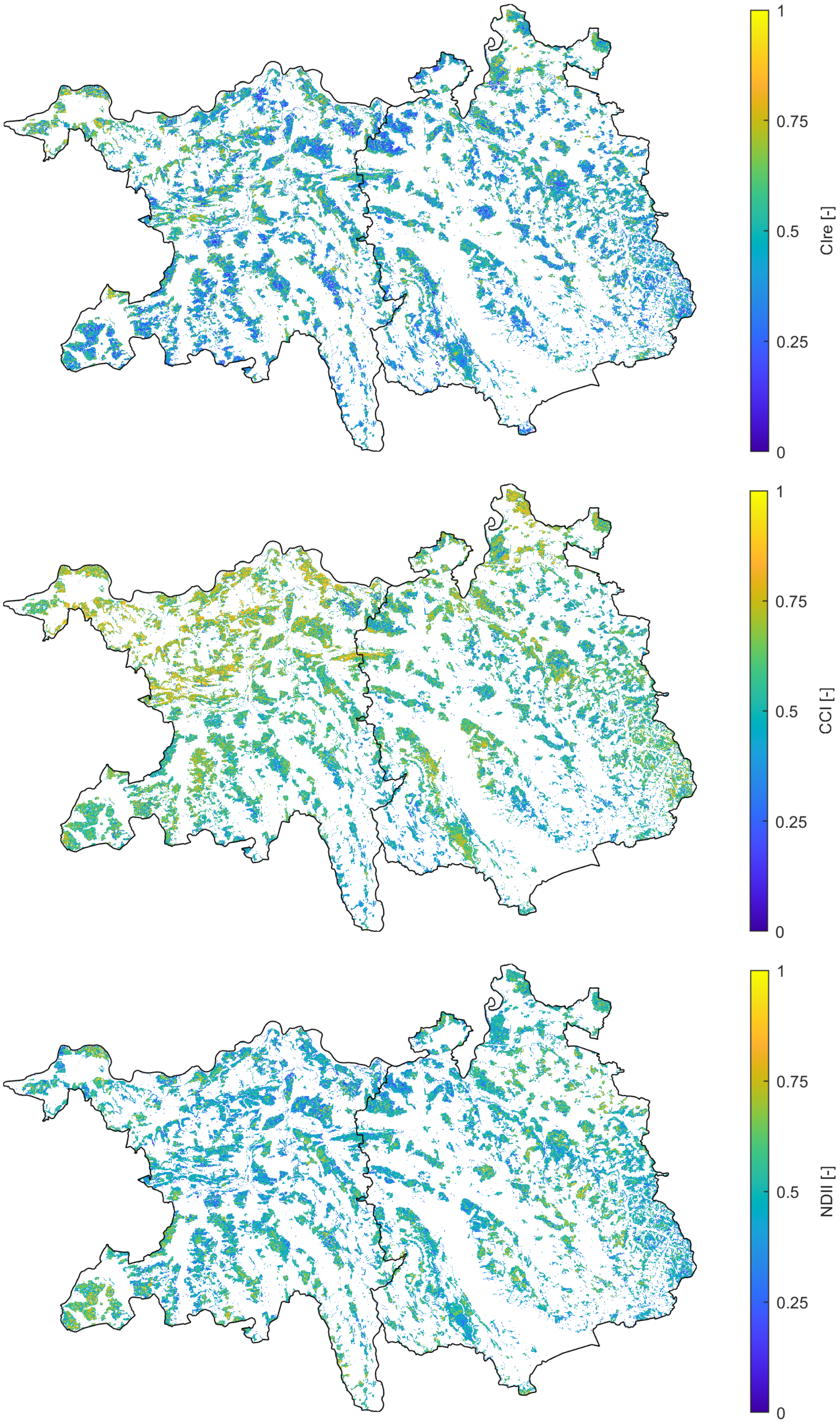

Figure S3: Calculated indices CIre (top), CCI (middle), and NDII (bottom) as proxies for the physiological traits CHL, CCR, and EWT at the research site and normalized between 0 and 1. The RGB composite of the three traits is illustrated in Figure S2.

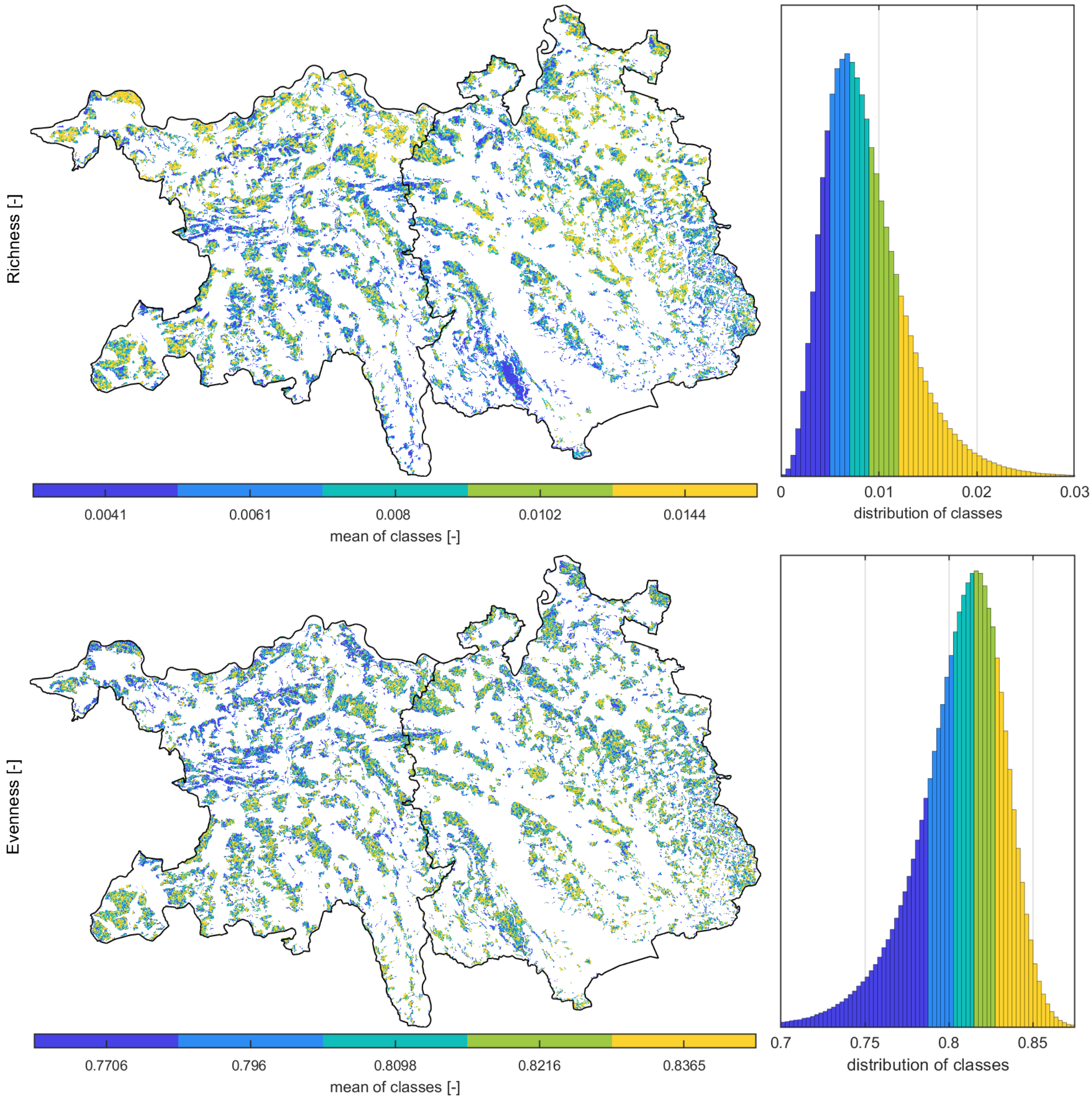

Figure S4: Functional richness and functional evenness maps of the study area (left) and histograms of distribution (right) calculated at a 60 m radius. The histogram colors indicate 20%-percentiles, with the mean of every class in the color bars. The histogram of richness is slightly skewed toward zero, and richness varies between zero and 0.03. Evenness varies between 0.7 and 0.9, with a histogram skewed towards 1. The richness and evenness map showed a correlation coefficient of r = 0.027.

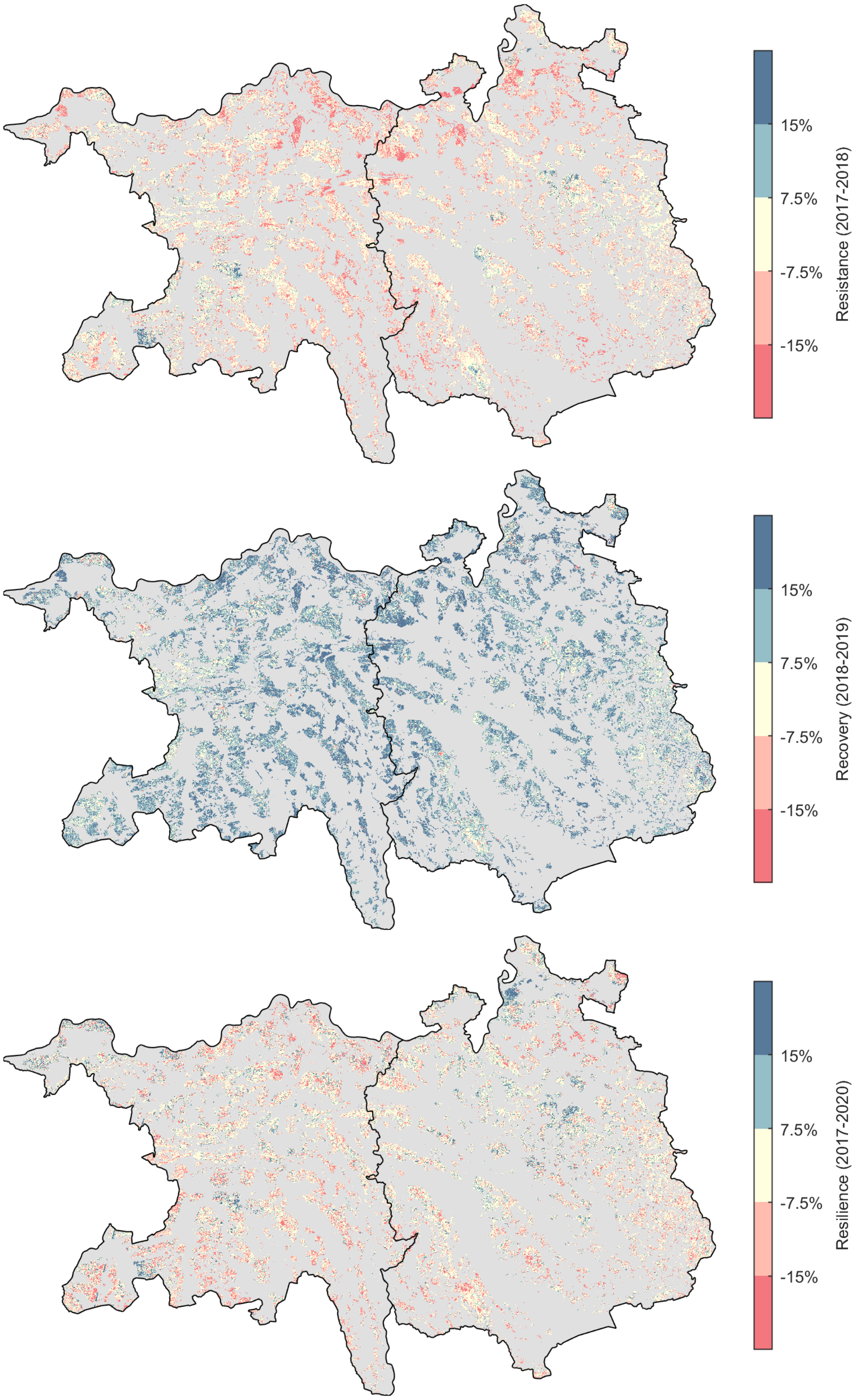

Figure S5: Drought response of forest ecosystems. NDWI-based drought response for the forested area in August composites for 2017–2020. The drought response is quantified using resistance (top, difference 2017–2018 in percent of 2017), recovery (center, difference 2018–2019 in percent of 2018), and resilience (bottom, difference 2017–2020 in percent of 2017). The mean resistance was -6.03%, mean recovery was 15.19% and resilience was -2.18%.

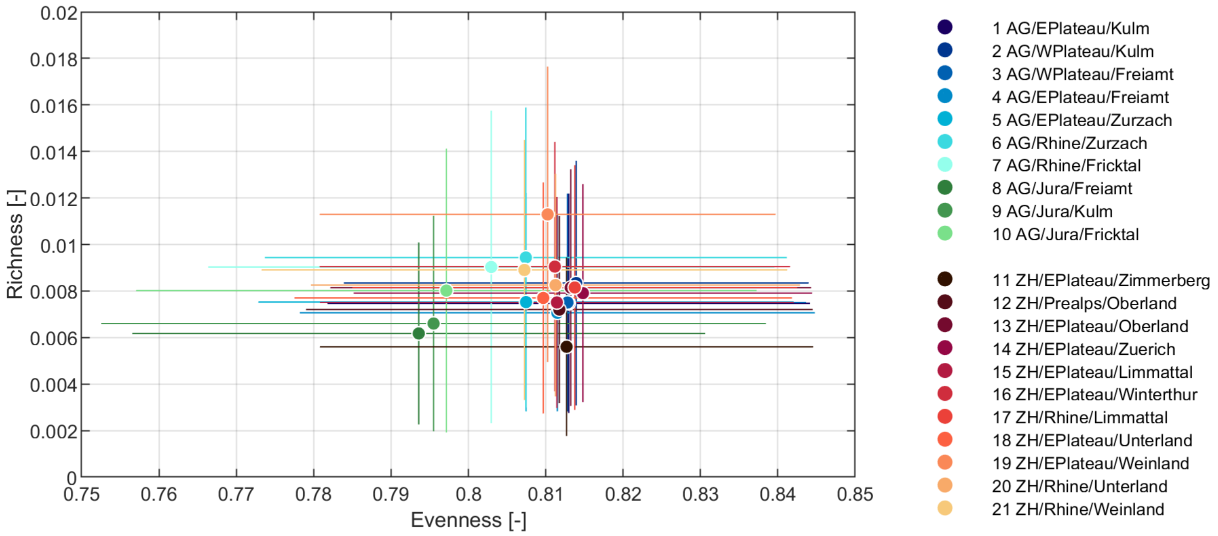

Figure S6: Average diversity of 21 subregions showing their mean richness and evenness and their quartile ranges. This graph illustrates that the variation within the regions is large, and the differences between regions are comparatively small. The subregions shown on the right were obtained by grouping the forests of the study area according to the intersection of 1) canton (Aargau (AG) and Zurich (ZH)), 2) geographical regions (Central Plateau (Eastern & Western), Rhine plains, Jura, and Pre-Alps), and 3) four, respectively seven, cantonal forest districts. Blue-green colors represent canton AG, and red-yellow colors represent canton ZH. The color gradients range from southern to northern regions within cantons.

Table S1: Area of forest response strength to the drought of 2018. Resistance, recovery, and resilience were divided into five classes, from strongly negative ($< -15\%$) to strongly positive ($> 15\%$) changes of the Normalized Difference Water Index (NDWI). The percentage of forest area falling into each class for each drought response measure is indicated.

Change	$< -15\%$	$< -7.5\%$	$-7.5\% - 7.5\%$	$> 7.5\%$	$> 15\%$	Total
Resistance	16.15%	22.47%	53.80%	5.53%	2.05%	100%
Recovery	1.50%	1.37%	23.62%	33.13%	40.38%	100%
Resilience	12.15%	16.34%	54.05%	10.92%	6.53%	100%

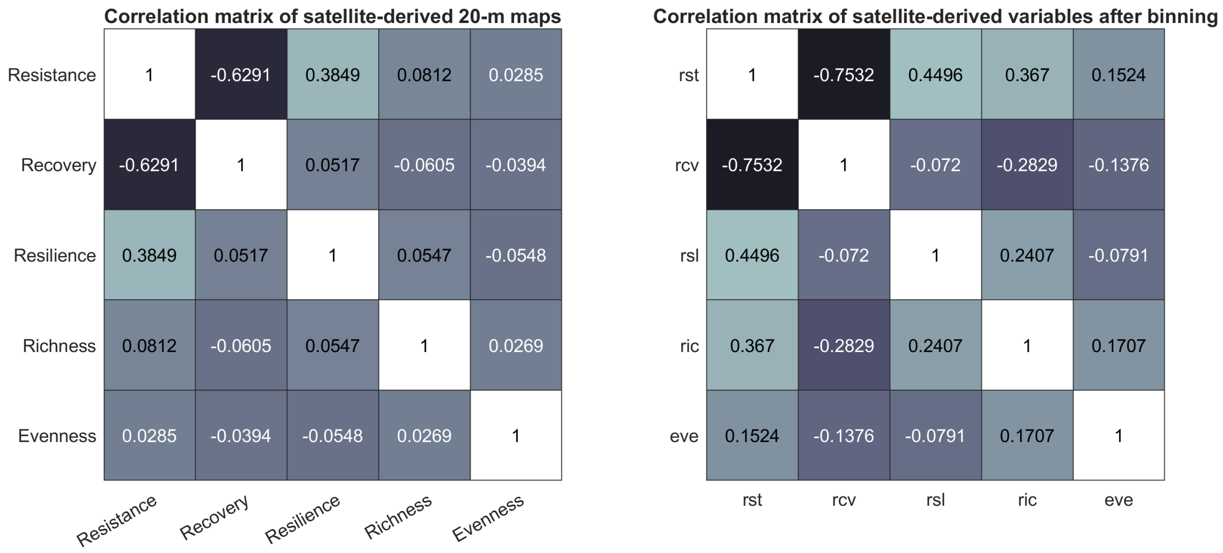

Figure S7: Left: Correlation matrix of the satellite-derived maps (Resistance, Recovery, Resilience, Richness, Evenness). Right: Correlation matrix of the resulting variables after binning into 20 by 20 bins (*ric*, *eve*, *rst*, *rcv*, *rsl*) as used in the linear models. Resistance and recovery show a negative correlation. Resistance and resilience show moderate positive correlation. A weak correlation was observed between the maps used in the models.

Table S2: Analysis of variance for resistance as dependent variable and diversity metrics and region as explanatory terms. logric = log-transformed richness, eve = evenness, eve2 = evenness squared, REG = region, Df = degree of freedom, SS = sum of squares (in thousands), %SS = SS in percent (corresponding to increments of model multiple r2 * 100), MS = mean square, F1 = F-ratio using MS of residuals as denominator, F2 = F-ratio using MS of interaction with region as denominator (this corresponds to a mixed-model analysis with the interaction as random-effects term). All F1 were highly significant (p <0.001), for F2 significances are indicated by asterisks (*** p <0.001, * p <0.05).

Response:	rst					
	Df	SS/1000	%SS	MS/1000	F1	F2
logric	1	2744	11.38	2744	5237	40.6***
eve	1	201	0.83	201	384.5	5.9*
eve2	1	509	2.11	509	970.7	85.9***
REG	20	15240	63.22	762	1454.4	
logric x REG	20	1352	5.61	68	129.1	
eve x REG	20	678	2.81	34	64.7	
eve2 x REG	20	119	0.49	6	11.30	
Residuals	6232	3265	13.54	0.5		
Total	6315	24108	100			
		r^2	0.865			

Table S3: Analysis of variance for recovery as dependent variable and diversity metrics and region as explanatory terms. logric = log-transformed richness, eve = evenness, eve2 = evenness squared, REG = region, Df = degree of freedom, SS = sum of squares (in thousands), %SS = SS in percent (corresponding to increments of model multiple r2 * 100), MS = mean square, F1 = F-ratio using MS of residuals as denominator, F2 = F-ratio using MS of interaction with region as denominator (this corresponds to a mixed-model analysis with the interaction as random-effects term). All F1 were highly significant (p <0.001), for F2 significances are indicated by asterisks (*** p <0.001).

Response:	rcv					
	Df	SS/1000	%SS	MS/1000	F1	F2
logric	1	5331	13.40	5331	3119.8	61.2***
eve	1	1251	3.14	1251	732.1	19.5***
eve2	1	724	1.82	724	423.8	37.5***
REG	20	18421	46.30	921	539	
logric x REG	20	1744	4.38	87	51	
eve x REG	20	1284	3.23	64	37.6	
eve2 x REG	20	385	0.97	19	11.3	
Residuals	6232	10650	26.77	1.7		
Total	6315	39790	100			
		r^2	0.732			

Table S4: Analysis of variance for resilience as dependent variable and diversity metrics and region as explanatory terms. ric = richness, eve = evenness, eve2 = evenness squared, REG = region, Df = degree of freedom, SS = sum of squares (in thousands), %SS = SS in percent (corresponding to increments of model multiple r2 * 100), MS = mean square, F1 = F-ratio using MS of residuals as denominator, F2 = F-ratio using MS of interaction with region as denominator (this corresponds to a mixed-model analysis with the interaction as random-effects term). All F1 were highly significant (p <0.001), for F2 significances are indicated by asterisks (*** p <0.001).

Response:	rsl					
	Df	SS/1000	%SS	MS/1000	F1	F2
ric	1	1582	8.43	1582	2027.8	19.2***
eve	1	1692	9.01	1692	2168.8	45.9***
REG	20	8239	43.89	412	528.2	
ric x REG	20	1647	8.77	82	105.6	
eve x REG	20	737	3.93	37	47.2	
Residuals	6253	4877	25.98	0.8		
Total	6315	18774	100			
		r^2	0.740			

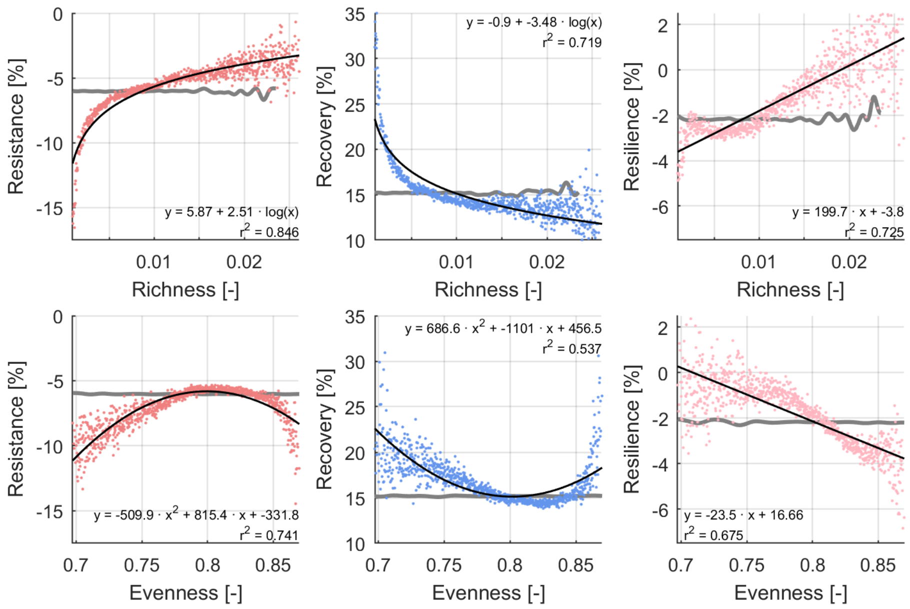

Figure S8: Resistance, recovery, and resilience (left to right) binned to 1000 bins of richness (top) and evenness (bottom) calculated at a 60 m radius. The black line represents the best-fit function. The gray line shows the null model of the experiment (all trait values shuffled prior to the calculation).

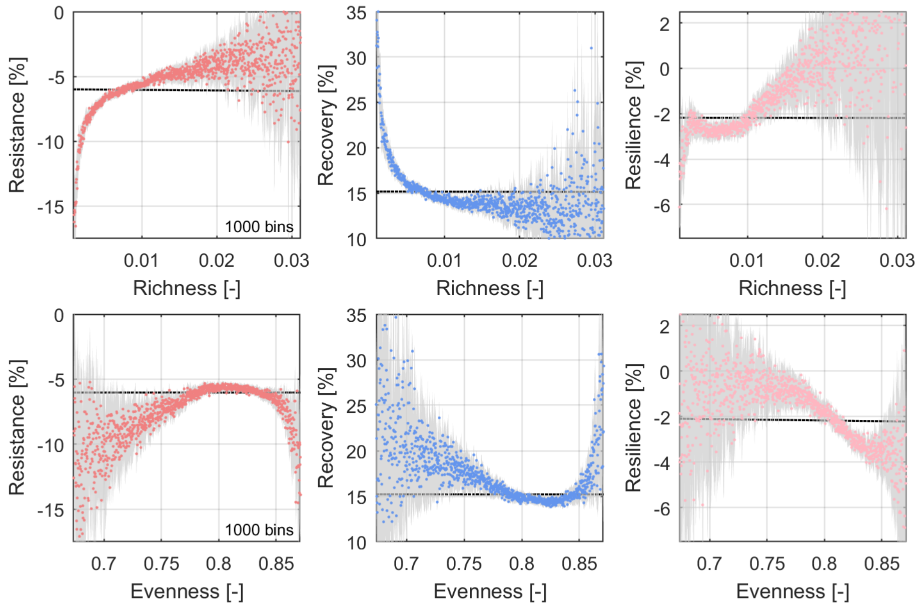

Figure S9: Resistance, recovery, and resilience (left to right) binned to 1000 bins of functional richness (top) and functional evenness (bottom) calculated at a 60 m radius. Empty or small bins are included in the graph, showing high variability within the bins. The gray area represents the 99% confidence interval.

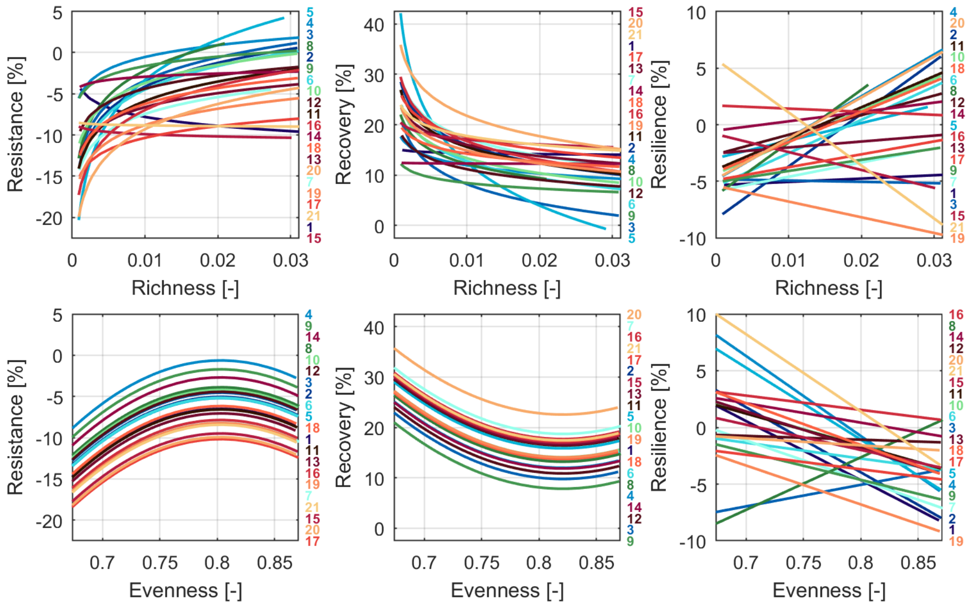

Figure S10: Regional drought responses resistance, recovery and resilience (left to right) as functions of functional richness (top) and functional evenness (bottom) calculated at a 60 m radius.

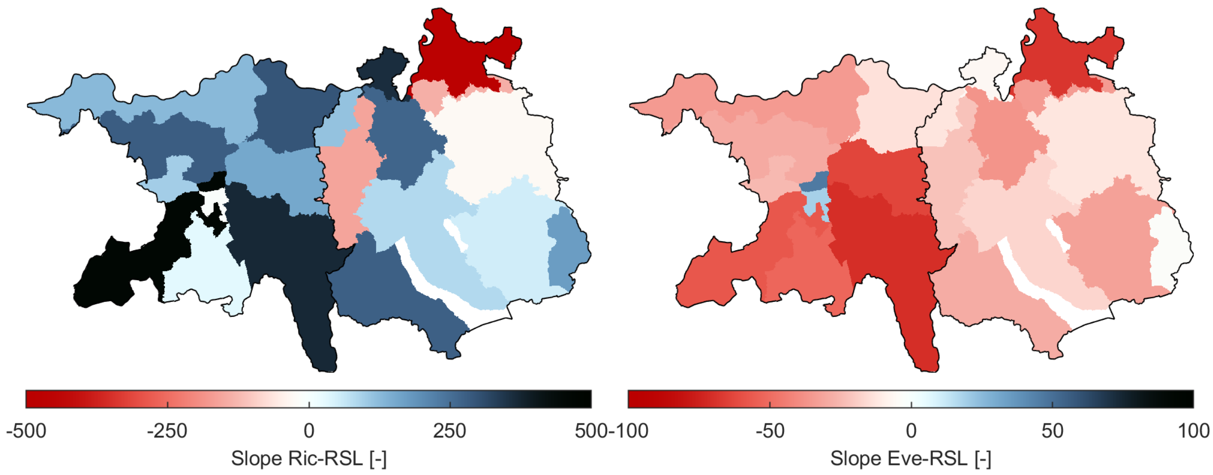

Figure S11: Regional slopes of resilience (RSL) as a function of functional richness (Ric) (left) and functional evenness (Eve) (right). Blue colors represent increasing slopes, red colors represent decreasing slopes.

S1 Validation of the drought resilience maps

To validate the 2020 resilience approach, we prepared a reference dataset with 271 data points representing 20-m Sentinel-2 pixels for the Sihlwald region. The Sihlwald is a 1098-ha natural reserve, ranging from 483 to 866 m a.s.l. in the southeast of the study area Brändli *et al.* (2020). Each 20-m pixel was optically evaluated for damage in the canopy and classified as damaged or intact in 2020 and unharmed in 2018. Sihlwald reported damage without any management cuttings, which excludes potential bias due to the removal of damaged trees with the potential to recover in the seasons between 2018 and 2020. The only exception is around pathways and roads to minimize the risk of falling trees for visitors and traffic. The park data were based on the forest inventory from 1990 (GIS Wildnispark Zürich & Grün Stadt Zürich, 1990).

We created the validation dataset using aerial images RGB/infrared from summer 2018/2020 provided by the canton Zurich GIS-ZH (2018, 2020). The 2018 dataset was acquired in the Sihlwald area between 27 July 2018 and 3 August 2018 on two dates. The 2020 data were acquired on three days between 9 and 12 August 2020. Both images were resampled to 0.5 m. Using high-resolution optical data gave clear advantages over identifying crown damage in the field. Data digitized by the canton allowed us to locate the pixels containing the canopy unambiguously, and we could see damage to the top layer of the forest, which might not necessarily have been visible from the ground in the forest.

We labeled intact and damaged satellite pixels by interpreting a pre-selection through high-resolution images of the area of interest. An example of this method is shown in Figure S12. The pre-selection was done by calculating the mean μ and standard deviation σ of the change in NDVI for the pixels that showed NDVI values of > 0.4 in 2018. Satellite pixels needed a minimum of 75% healthy forest pixels in 2018. Pixels showing a negative change of $< 2\sigma$ from the mean change were pre-classified as 'damaged,' and pixels showing a positive, neutral, or negative change $> \sigma$ from the mean change in NDVI were classified as 'intact.' We ended up with a pre-selection of 649 damaged and 2834 intact pixels. We selected the pixels in a random sampling for optical selection of the validation dataset from 200 pixels per class, regularly distributed along the test site. We optically decided if the canopy showed significant ($>$ 50% of the pixel area) damage to the canopy in 2020 or was optically intact and healthy. The criteria to be selected for the reference were an intact canopy in 2018 and, if visible, no roads in proximity. Additionally,

the canopy should be visible, with no large-area shadow effects or similar in either year. Based on these criteria, we selected 150 damaged and 121 intact pixels, which were used to validate the 2020 drought resilience. We validated using a standard confusion matrix with dead pixels classified as having resilience $< 15\%$ and a t-test with continuous resilience values.

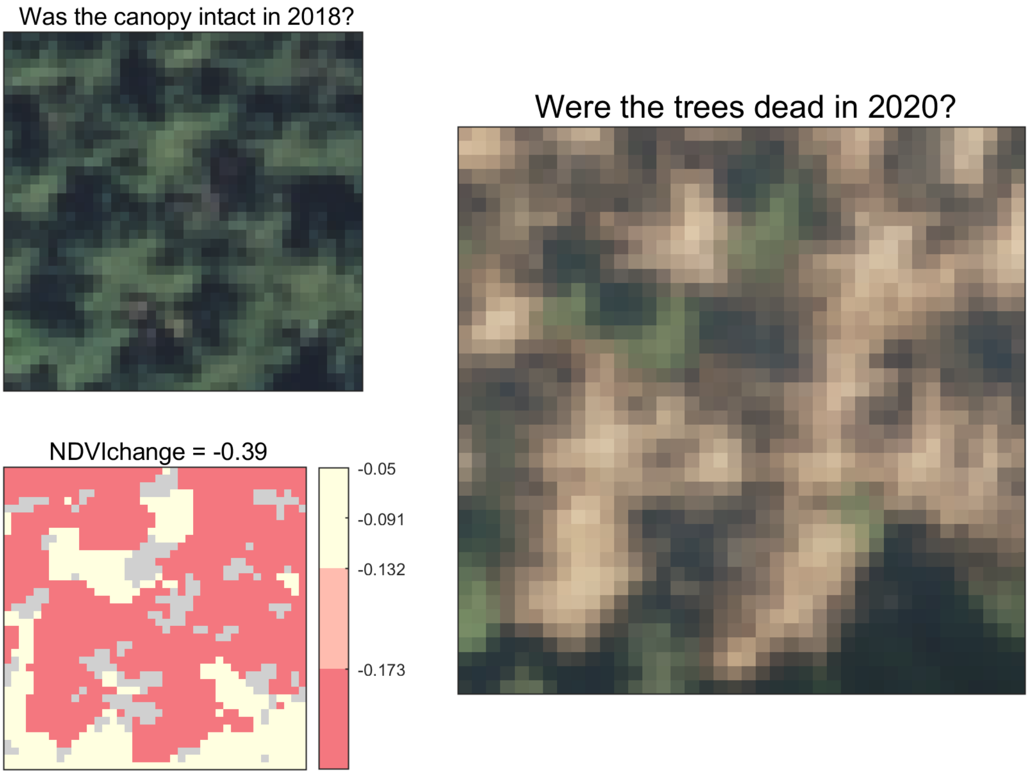

Figure S12: Graphical representation of a pre-selected pixel as displayed for optical selection. The pixel shown here was classified as 'damaged' in pre-selection and the optical selection processes. The requirements for the classification were an intact canopy in 2018 and evident damage to the canopy in 2020. Furthermore, the same section should be identifiable and recognizable without, for example, overly large shadows.

From a pre-selection based on NDVI values, groups of damaged and non-damaged ('intact') areas were identified in 2020 compared to 2018. Visually damaged areas showed a different drought response than non-damaged areas. Welch's t-test indicates a significant difference between the groups (Figure S13). For damaged pixels, we achieved a user's accuracy of 97.26% and a producer's accuracy of 94.67%. The user's accuracy for intact pixels was 97.87%, and the producer's accuracy was 76.03%. The comparably low producer's accuracy for intact pixels can be explained by the validation dataset, including visible damage. Trees that suffered greatly during the drought might show a reduction in water content and LAI but might not show visible damage in the validation dataset.

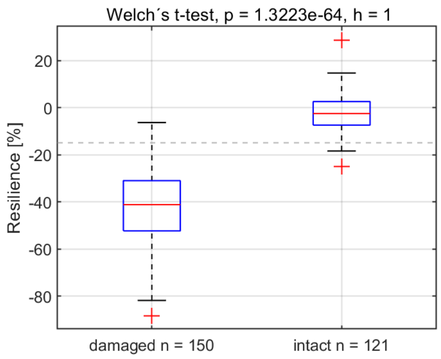

Figure S13: Boxplots showing the validation results for the two classes 'damaged' and 'intact.'

S2 Multi-scale analysis

We tested for scale effects using different radii (60 m, 120 m, and 240 m) to derive diversity metrics, resulting in different calculation areas ranging from 1.1 ha to 18.1 ha (see Supplementary Table S5). The three calculation radii were selected as approximations for the calculations in 100 m, 250 m, and 500 m, which were assumed to be relatively large ecosystem scales and landscape scales Oehri *et al.* (2020); Zhang *et al.* (2018); Zheng *et al.* (2023).

Table S5: The three different calculation radii and resulting area.

Radius	60 m	120 m	240 m
# Pixels	28.3 p	113.1 p	452.39 p
Area	1.131 ha	4.524 ha	18.096 ha

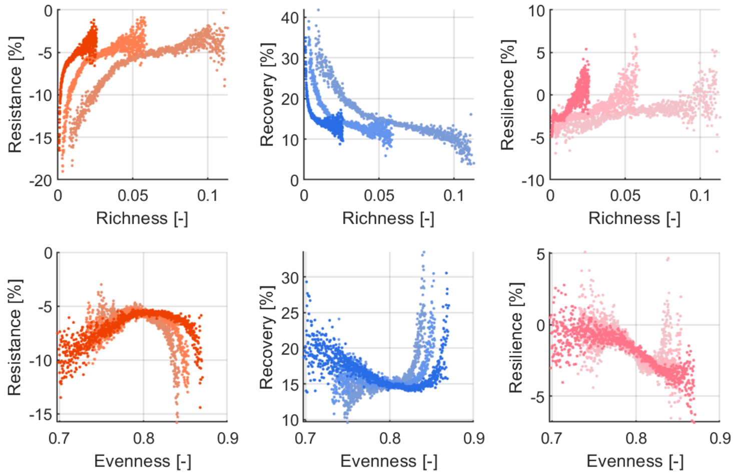

Figure S14: Change in RST, RCV, and RSL by quantiles of functional richness (top) and functional evenness (bottom) defined classes with absolute mean values of respective class and by calculation area (1.1 ha – 4.5 ha, dark to light color). The values of diversity ranking (mean per bin) are sorted from low to high.

Figure S14 shows the change in drought response with trait-based diversity at the three different scales of calculation 1.13 ha, 4.5 ha, and 18 ha. Functional richness results in higher values when derived from a larger calculation area, as it is directly affected by the number of data points Karadimou *et al.* (2016). Although the value ranges change, the qualitative relationship is constant across the scales. Functional evenness shows a smaller range of values at a large grid. The evenness–resistance relationship shows a less clear hump shape at larger scales, mainly due to the lower value range of low evenness values. Besides these observations, resilience shows a clear relationship with evenness at all calculation scales. However, this relationship is less clear due to the smaller value range and more outliers.

Supplementary References

Brändli K., Stillhard J., Hobi M. & Brang P. (2020) Waldinventur 2017 im Naturerlebnispark Sihlwald. *WSL Berichte*, **93**, 52.

GIS-ZH (2018) Orthofoto Sommer RGB/Infrarot 2018 (OGD). URL https://www.geolion.zh.ch/geodatensatz/show?gdsid=493.

GIS-ZH (2020) Orthofoto Sommer RGB/Infrarot 2020 (OGD). URL https://www.geolion.zh.ch/geodatensatz/show?gdsid=527.

GIS Wildnispark Zürich & Grün Stadt Zürich (1990) Waldbestandesaufnahme Sihlwald 1990. URL https://www.parcs.ch/wpz/mmd_fullentry.php?docu_id=9075.

Karadimou E.K., Kallimanis A.S., Tsiripidis I. & Dimopoulos P. (2016) Functional diversity exhibits a diverse relationship with area, even a decreasing one. *Scientific Reports*, **6**, 35420. https://doi.org/10.1038/srep35420.

Oehri J., Schmid B., Schaepman-Strub G. & Niklaus P.A. (2020) Terrestrial land-cover type richness is positively linked to landscape-level functioning. *Nature Communications*, **11**, 1–10. https://doi.org/10.1038/s41467-019-14002-7.

Zhang H., Chen H.Y.H., Lian J., John R., Ronghua L., Liu H., Ye W., Berninger F. & Ye Q. (2018) Using functional trait diversity patterns to disentangle the scale-dependent ecological processes in a subtropical forest. *Functional Ecology*, **32**, 1379–1389. https://doi.org/10.1111/1365-2435.13079.

Zheng Z., Schmid B., Zeng Y., Schuman M.C., Zhao D., Schaepman M.E. & Morsdorf F. (2023) Remotely sensed functional diversity and its association with productivity in a subtropical forest. *Remote Sensing of Environment*, **290**, 113530. https://doi.org/10.1016/j.rse.2023.113530.
